# Transient inhibition of type I interferon enhances CD8^+^ T cell stemness and vaccine protection

**DOI:** 10.1084/jem.20241148

**Published:** 2025-03-10

**Authors:** Benjamin J. Broomfield, Chin Wee Tan, Raymond Z. Qin, Hanna Abberger, Brigette C. Duckworth, Carolina Alvarado, Lennard Dalit, Chee Leng Lee, Rekha Shandre Mugan, Zihnil A.I. Mazrad, Hiromi Muramatsu, Liana Mackiewicz, Bailey E. Williams, Jinjin Chen, Asuka Takanashi, Stewart Fabb, Marc Pellegrini, Kelly L. Rogers, Woohyun J. Moon, Colin W. Pouton, Melissa J. Davis, Stephen L. Nutt, Norbert Pardi, Verena C. Wimmer, Joanna R. Groom

**Affiliations:** 1 Walter and Eliza Hall Institute of Medical Research, Parkville, Australia; 2 Department of Medical Biology, The University of Melbourne, Parkville, Australia; 3 Frazer Institute, Faculty of Medicine, The University of Queensland, Brisbane, Australia; 4 Drug Delivery, Disposition and Dynamics, Monash Institute of Pharmaceutical Sciences, Monash University, Parkville, Australia; 5 Department of Microbiology, Perelman School of Medicine, Philadelphia, PA, USA; 6 Centenary Institute of Cancer Medicine and Cell Biology, Camperdown, Australia; 7 Acuitas Therapeutics, Vancouver, Canada; 8 School of Biomedicine, Faculty of Health and Medical Sciences, The University of Adelaide, Adelaide, Australia

## Abstract

Developing vaccines that promote CD8^+^ T cell memory is a challenge for infectious disease and cancer immunotherapy. TCF-1^+^ stem cell–like memory CD8^+^ T (T_SCM_) cells are important determinants of long-lived memory. Yet, the developmental requirements for T_SCM_ cell formation are unclear. Here, we identify the temporal window for type I interferon receptor (IFNAR) blockade to drive T_SCM_ cell generation following viral infection and mRNA–lipid nanoparticle vaccination. We reveal a reversible developmental trajectory where transcriptionally distinct T_SCM_ cells emerged from a transitional precursor of exhausted T cellular state concomitant with viral clearance. T_SCM_ cell differentiation correlated with T cell retention within the lymph node paracortex due to disrupted CXCR3 chemokine gradient formation. These effects were linked to increased antigen load and a counterintuitive increase in IFNγ, which controlled cell location. Vaccination with the IFNAR blockade promoted T_SCM_ cell differentiation and enhanced protection against chronic infection. These findings propose an approach to vaccine design whereby modulation of inflammation promotes memory formation and function.

## Teaser

Broomfield et al. demonstrate that early type I interferon blockade alters CXCR3 chemokine regulation and cell location to promote an antigen-dependent CD8^+^ T cell transition of precursor of exhausted T cells to stem cell–like memory CD8^+^ T cells for enhanced protection following viral infection and mRNA–lipid nanoparticle vaccination.

## Introduction

Vaccines that promote and sustain CD8^+^ T cell memory are an ongoing challenge for infectious disease and cancer immunotherapy. TCF-1^+^ stem cell–like memory CD8^+^ T (T_SCM_) cells have emerged as important determinants of long-lived T cell memory (Gattinoni et al., 2017; Zhao et al., 2022). The induction and maintenance of T_SCM_ cells is a characteristic of vaccines regarded as exemplars of long-lived immunity (Ahmed and Akondy, 2011; Akondy et al., 2017; Hagan et al., 2022; Pais Ferreira et al., 2020). The protective potential of T_SCM_ cells lies in their long-term persistence, high proliferative capacity, and ability to generate effector CD8^+^ T (T_EFF_) cells upon rechallenge (Akondy et al., 2017; Fuertes Marraco et al., 2015; Gattinoni et al., 2011; Jeannet et al., 2010; Pais Ferreira et al., 2020; Zhou et al., 2010). In chronic infections and cancer, a population of exhausted cells, known as precursors of exhausted CD8^+^ T (T_PEX_) cells, are marked by TCF-1 expression and are thought to be analogous to the T_SCM_ cell population identified following vaccination and acute infection (Im et al., 2016; Utzschneider et al., 2016; Wu et al., 2016; Zhou et al., 2010). T_PEX_ cells are the major cell type responding to immune checkpoint blockade (Beltra et al., 2020; Huang et al., 2022; Miller et al., 2019; Siddiqui et al., 2019), and their numbers are predictive of patient outcome (Huang et al., 2017; Sade-Feldman et al., 2018; Thommen et al., 2018). Given the demonstrated importance of each of these TCF-1^+^ stem-like cell populations for infectious disease protection and cancer immunotherapy, understanding their developmental relationship and how their generation can be specifically directed could enhance vaccine-induced protection and immunotherapy (Held et al., 2019; Lugli et al., 2020; Zhao et al., 2022).

A key determinant of CD8^+^ T cell differentiation is the site-specific environmental cues, which guide the transcriptional regulators of the T cell fate (Duckworth et al., 2022; Farsakoglu et al., 2021). Several studies have leveraged cytokine cues to re-invigorate existing populations of stem-like CD8^+^ T cells in chronic settings via targeted delivery of IL-2 and CD4^+^ T cell–derived IL-21 (Beltra et al., 2022, *Preprint*; Codarri Deak et al., 2022; Hashimoto et al., 2022; Li et al., 2021; Snell et al., 2018; Tichet et al., 2023; Zander et al., 2019), or use of IL-7 and IL-15 to optimize in vitro differentiation of T_SCM_-like cells for chimeric antigen receptor T cell therapy (Cieri et al., 2013; Meyran et al., 2023). In contrast, less is known about directing T_SCM_ cell differentiation for durable CD8^+^ T cell immunity. T_SCM_ cells arise early during an inflammatory response, alongside the differentiation of T_EFF_ cells (Broomfield and Groom, 2024; Chan et al., 2024; Duckworth et al., 2021; Pais Ferreira et al., 2020). Within lymph nodes, the migration of CD8^+^ T cells into distinct niches is underwritten by transcriptional control of chemokine receptors (Duckworth and Groom, 2021). The expression of *Tbx21* (encoding T-bet) regulates CXCR3 expression and migration to the lymph node interfollicular regions (IFRs) to imprint the T_EFF_ cell fate (Duckworth et al., 2021). Stem-like T cell differentiation is instructed by TCF-1 (encoded by *Tcf7*), which regulates CCR7 expression to retain cells in the T cell paracortex (Duckworth et al., 2021; Im et al., 2016). Thus, T_SCM_ cell differentiation occurs in distinct microenvironments to that of T_EFF_ cells (Duckworth et al., 2022; Pham et al., 2009). However, how chemokine gradients are regulated to position newly activated CD8^+^ T cells is unclear.

Interferons (IFNs) are potent antiviral cytokines that belong to three major families (Lazear et al., 2019). The timing and location of the IFN response to viral infection are tightly coordinated (Sposito et al., 2021). Dysregulation of type I IFN (IFN-I) or their signaling pathways can exacerbate viral diseases such as influenza, measles, herpes simplex virus infection, and coronavirus disease 2019 (Bastard et al., 2020; Casanova and Abel, 2022; Hernandez et al., 2019; Zhang et al., 2020; Zhang, 2020). Although this demonstrates IFN-Is are essential for control of viral infection, chronic induction of IFN-I counteracts positive immune responses to potentiate immune dysfunction (Cheng et al., 2017; Crouse et al., 2015; Marx et al., 2023; Teijaro et al., 2013; Wilson et al., 2013; Wu et al., 2016). In chronic infection, blocking IFN-I receptor (IFNAR) signaling directs T_PEX_ cell differentiation to reduce persistent viral load (Cheng et al., 2017; Marx et al., 2023; Teijaro et al., 2013; Wu et al., 2016). In contrast to the protective role of IFN-I during acute viral infection, the expression of the only type II IFN (IFN-II) family member, IFNγ, is a biomarker for severe disease, and in combination with TNF-α can induce cell death, leading to severe tissue damage and immunopathology (Alspach et al., 2019; Karki et al., 2021; Sposito et al., 2021; Walker et al., 2021).

IFNs exert their pleiotropic immunomodulatory effects by inducing IFN-stimulated genes (ISGs) in multiple cell types. Although the ligands of CXCR3—CXCL9, CXCL10, and CXCL11—are key ISGs (Groom and Luster, 2011a, 2011b; Marsman et al., 2018), it is unknown how blocking IFNAR during viral infection impacts chemokine expression for preferential formation of T_SCM_ cells. Indeed, there is a considerable overlap in the ISGs shared between distinct IFN families (Lazear et al., 2019; Rusinova et al., 2013; Walker et al., 2021). While the expression of CXCL9 and CXCL10 has been used as a surrogate for IFN-I expression, these ligands can be induced during acute and chronic viral infection or recall responses by both type I and II IFNs (Borriello et al., 2022; De Giovanni et al., 2020; Sung et al., 2012). Therefore, underexplored redundancy or compensation of IFNs may influence chemokine expression and, in turn, CD8^+^ T cell positioning and differentiation.

As the blockade of cytokine signals that usually promote the T_EFF_ cell fate, such as IL-12 and IFN-I, results in enhanced T_SCM_ cell differentiation (Danilo et al., 2018; Duckworth et al., 2021; Huang et al., 2019; Wu et al., 2016), we used temporal inhibition of IFNAR as a strategy to specifically enhance stem-like CD8^+^ T cell formation in the absence of T_EFF_ cell differentiation. Mechanistically, we established that T_SCM_ cell formation is preceded by a T_PEX_ cell state and established a method to monitor the transition and reversion of stem-like cellular states dependent on the presence or absence of antigen. T_SCM_ cell differentiation was regulated by an unappreciated IFN-I and IFN-II interplay that augments CXCR3 chemokine expression and T cell location. In combination with mRNA–lipid nanoparticle (mRNA-LNP) vaccination, we demonstrate that IFNAR blockade results in enhanced vaccine efficacy and immune protection, thus revealing a tractable strategy to drive potent CD8^+^ T cell memory.

## Results

### Early, short-term inhibition of IFN-I optimizes T_SCM_ cell differentiation

**Figure 1.**
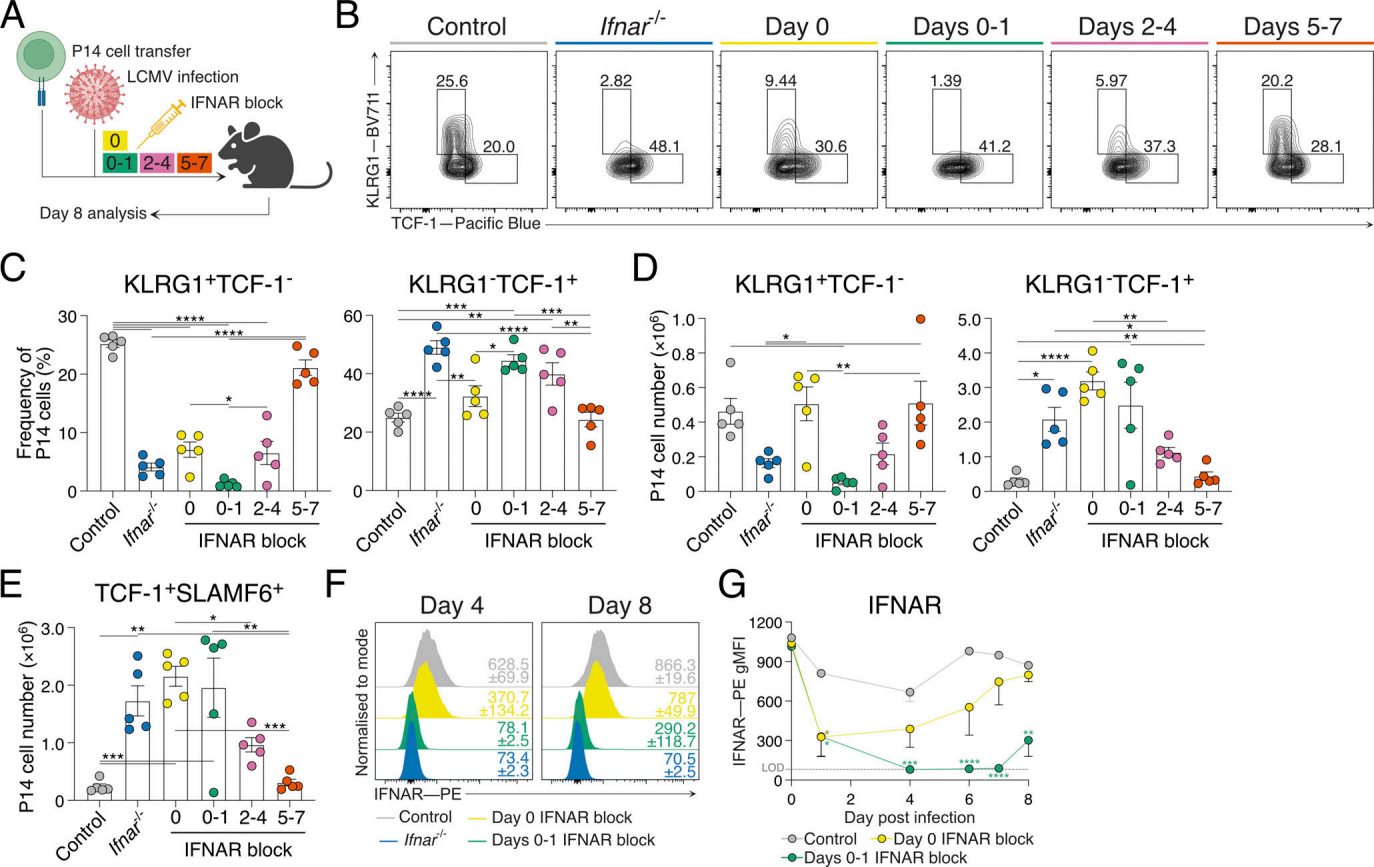
**IFNAR blocking at d0–1 of acute LCMV infection directs stem-like T cell differentiation. (A–E)** Analysis of P14 cells generated in groups indicated in A. Data are representative of two independent experiments with five mice per group in each experiment. Each dot in C–E represents a single mouse. Data are the mean ± SEM. Statistical differences were analyzed using one-way ANOVA tests. **(A)** Experimental scheme. P14 cells were transferred into wild-type hosts prior to infection with acute LCMV Armstrong and treated with indicated schedules (d0, d0–1, d2–4, d5–7) of IFNAR blocking monoclonal antibodies. Peripheral lymph node P14 cells were analyzed at d8 of infection. **(B)** Representative flow cytometry plots of P14 cells showing KLRG1^+^TCF-1^−^ effector T (T_EFF_) and KLRG1^−^TCF-1^+^ stem-like T cell populations. **(C)** Graphs summarizing frequencies in B. **(D)** Graphs summarizing total P14 cell numbers of T_EFF_ and stem-like T cell subsets. **(E)** Graph summarizing total P14 cell number of TCF-1^+^SLAMF6^+^ stem-like T cells as shown in Fig. S1 A. **(F and G)** IFNAR detection of peripheral lymphocytes following acute LCMV infection and IFNAR blocking as indicated, or control *Ifnar*^*−/−*^ hosts. Data are representative of three independent experiments with four mice per group in each experiment. Average geometric mean fluoresence intensity (gMFI) ± SEM for each group are indicated. **(F)** Representative histograms of IFNAR expression. **(G)** Graph summarizing IFNAR gMFI. Statistical differences were analyzed using one-way ANOVA tests. The dashed line indicates anti-IFNAR staining LOD. *P < 0.05, **P < 0.01, ***P < 0.001, ****P < 0.0001. Fig. S1 shows additional supporting data. LOD, limit of detection.

**Figure S1.**
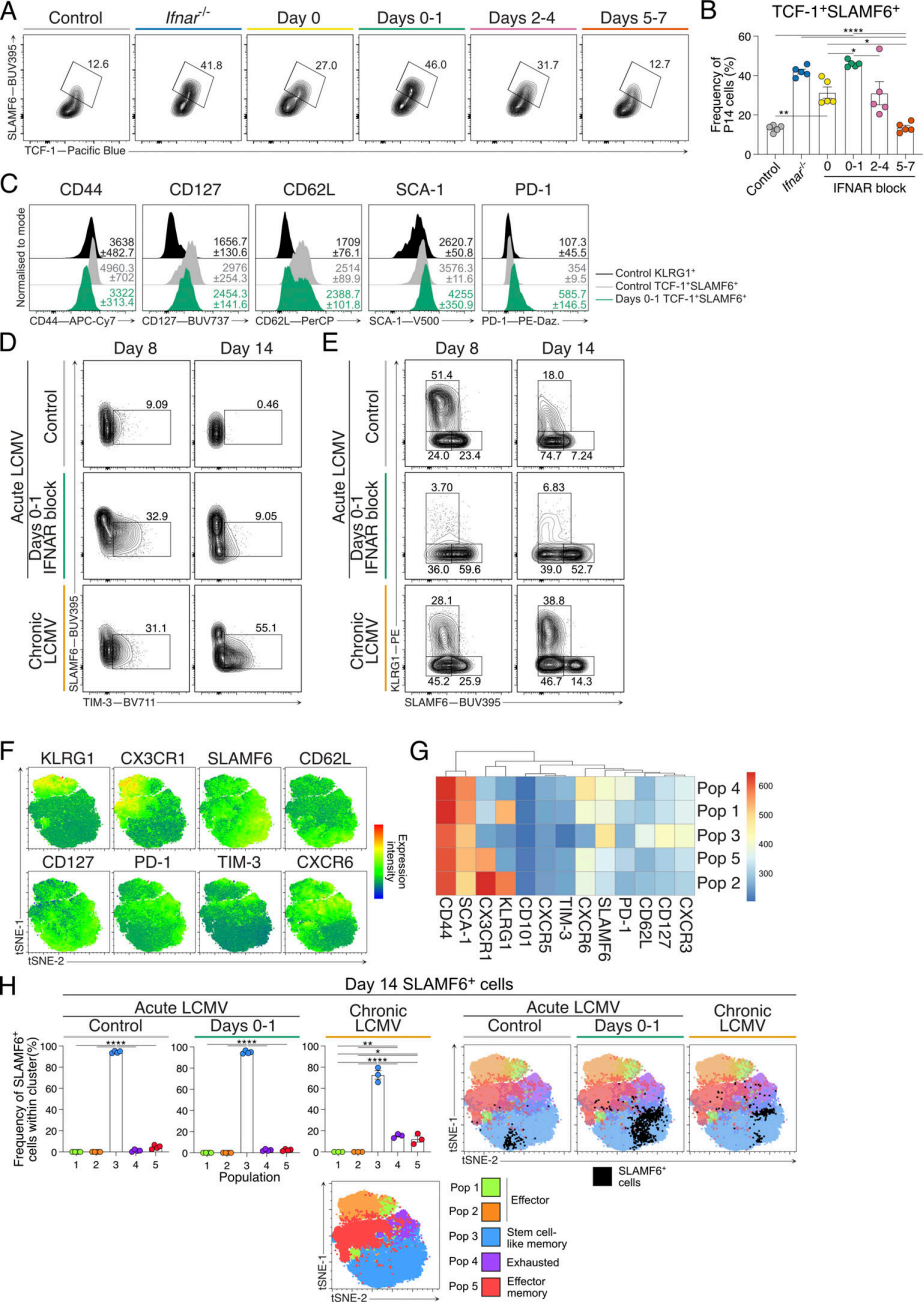
**IFNAR blocking at d0–1 of acute LCMV infection directs stem-like T cell differentiation without establishing chronic infection and exhaustion.** Related to Figs. 1 and 2. **(A–C)** P14 cells generated in groups indicated in Fig. 1 A. Data are representative of two independent experiments with five mice per group in each experiment. Each dot in B represents a single mouse. Data are the mean ± SEM. Statistical differences were analyzed using one-way ANOVA tests. Data are representative of three independent experiments with four mice per group in each experiment. Average gMFI ± SEM for each graph are indicated. **(A)** Representative plots of P14 cells showing stem-like (TCF-1^+^SLAMF6^+^) T cell populations. **(B)** Graph summarizing frequencies in A. **(C)** Representative histograms of T_EFF_ (KLRG1^+^; black histograms) and stem-like (TCF-1^+^SLAMF6^+^; gray histograms) P14 cell populations from treated control mice and stem-like (TCF-1^+^SLAMF6^+^; green histograms) P14 cells from d0–1 IFNAR-blocked mice for expression of CD44, CD127, CD62L, SCA-1, and PD-1. **(D–H)** Analysis of P14 cells from peripheral lymph nodes of mice at d8 or d14 of acute LCMV Armstrong with or without IFNAR block at d0–1, or chronic LCMV Docile infection. Data are representative of three independent experiments with four mice per group in each experiment. Each dot in H represents a single mouse. Data are the mean ± SEM. Statistical differences were analyzed using one-way ANOVA tests. **(D)** Representative plots of TIM-3 expression on P14 cells within each infection condition. **(E)** Representative flow cytometry plots of T_EFF_ (KLRG1^+^SLAMF6^−^) and stem-like (KLRG1^−^SLAMF6^+^) T cell populations within P14 cells from each group. **(F)** Overlay of marker expression heat maps on t-distributed stochastic neighbor embedding (tSNE) plot generated by FlowSOM. **(G)** FlowSOM heat map determining distinction of discreet populations. **(H)** Frequency of each FlowSOM population within d14 SLAMF6^+^ P14 cells for each infection condition, and corresponding representative overlay of SLAMF6^+^ P14 cells displayed in tSNE plots. *P < 0.05, **P < 0.01, ****P < 0.0001.

Deficiency in IFNAR or use of IFNAR blocking antibodies increases the frequency of memory CD8^+^ T cells (Duckworth et al., 2021; Huang et al., 2019; Lazear et al., 2019; Palacio et al., 2020; Wu et al., 2016). To understand the precise timing required to promote stem-like T cells, we tested how distinct IFNAR blocking schedules impacted antigen-specific P14 TCR-transgenic CD8^+^ T cell differentiation at the peak of the T cell response 8 days following intravenous acute lymphocytic choriomeningitis virus (LCMV) Armstrong infection (Fig. 1 A). Blocking at the time of infection and the following day (days 0 and 1, d0–1) led to the highest increase in the frequency and the number of TCF-1^+^SLAMF6^+^ stem-like T cells, which were formed in the near absence of KLRG1^+^ T_EFF_ cell differentiation (Fig. 1, B–E; and Fig. S1, A and B). In contrast, a single dose of IFNAR blocking at the time of infection (day 0, d0) promoted both stem-like and T_EFF_ cell formation (Fig. 1, B–E) (Palacio et al., 2020). Consistent with stem-like T cell differentiation, TCF-1^+^SLAMF6^+^ P14 cells in d0–1 IFNAR-blocked mice exhibited reduced CD44, and higher CD127, CD62L, SCA-1, and programmed cell death protein 1 (PD-1) expression, similar to the T_SCM_ cells observed in control-treated mice (Fig. S1 C).

To understand why d0–1 IFNAR blocking led to more directed T_SCM_ cell formation than treatment at the time of infection alone (d0), we investigated the duration of IFNAR receptor inhibition (Fig. 1, F and G). Competitive IFNAR epitope blocking was assessed by detecting binding of the same antibody clone on peripheral lymphocytes. While d0 treatment reduced anti-IFNAR binding immediately following infection, staining was regained by day 4 (d4) and steadily increased to the level of the control-treated mice, suggesting a single IFNAR blocking dose is insufficient to completely block the initial IFN-I burst following acute LCMV infection (Fig. 1, F and G). In contrast, in mice receiving d0–1 IFNAR block, IFNAR antibody staining remained close to the level of binding observed in *Ifnar*^*−/−*^ cells throughout infection, reflecting complete blockade of the IFNAR epitope (Fig. 1, F and G). Combined, our data identify the early temporal window for IFN-I blockade during acute LCMV infection to drive increased differentiation of TCF-1^+^ T_SCM_ cells.

### Early IFNAR blockade skews stem-like T cell differentiation without establishing chronic infection

**Figure 2.**
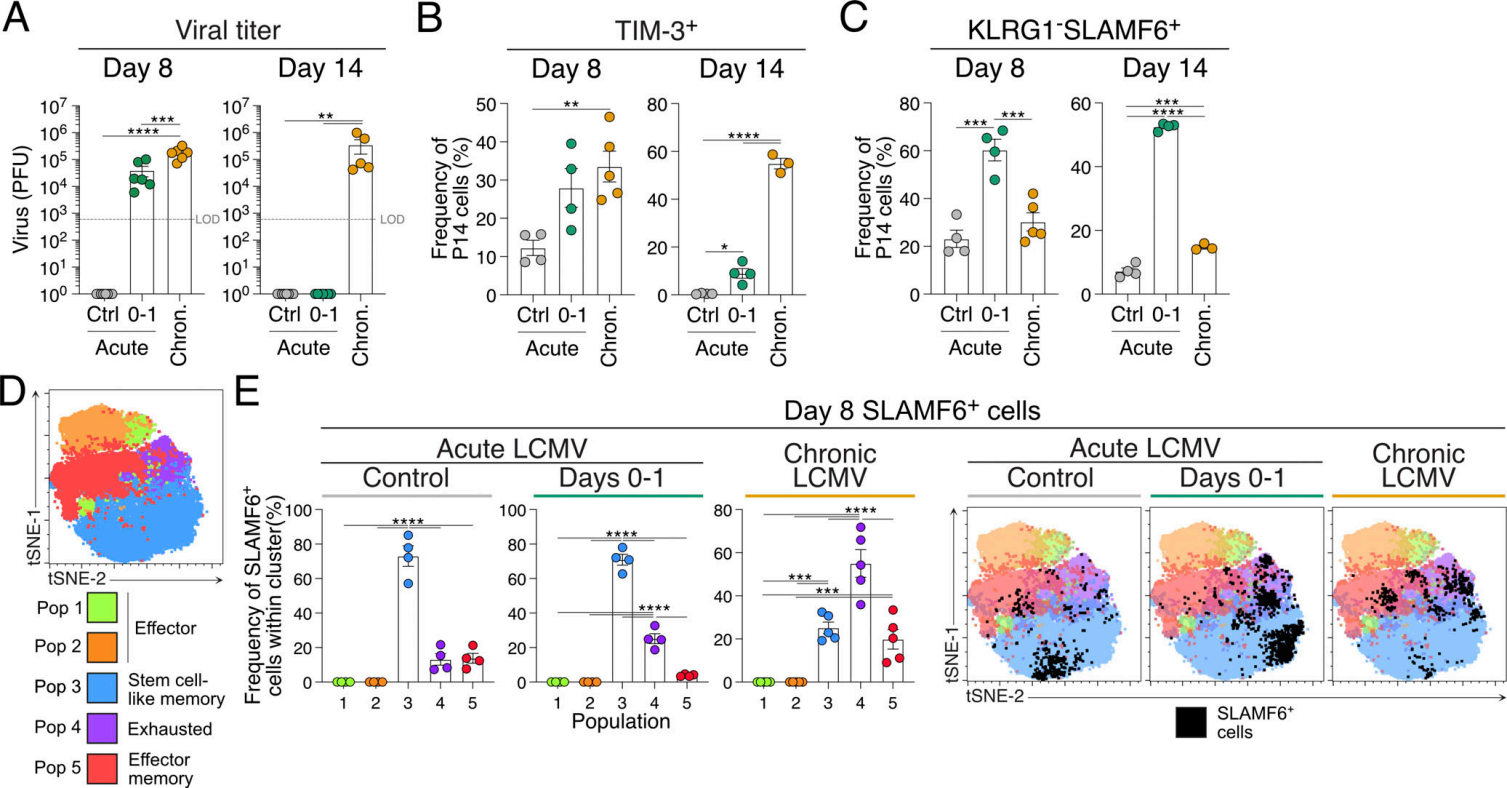
**Early IFNAR blocking skews stem-like T cell differentiation without establishing chronic infection and exhaustion. (A–E)** Analysis of P14 cells at d8 or d14 of acute LCMV infection, with or without IFNAR blocking at d0–1, or chronic LCMV Docile infection. Data are representative of three independent experiments with three to six mice per group in each experiment. Each dot in A–C and E represents a single mouse. Data are the mean ± SEM. Statistical differences were analyzed using one-way ANOVA tests. **(A)** Graphs summarizing the PFU from viable virus in spleens of mice in each infection condition. The dashed line indicates viral plaque LOD. **(B)** Graphs summarizing average frequencies of TIM-3 expression on P14 cells in each indicated group. **(C)** Graphs summarizing frequencies of stem-like cell populations within P14 cells in each group. **(D and E)** FlowSOM dimensionality reduction analysis of P14 cells generated in each infection condition. **(D)** FlowSOM dimensionality reduction analysis. tSNE plot displaying five FlowSOM-defined cell populations and denoted identities generated from differential surface antigen expression. **(E)** Frequency of each FlowSOM population within d8 SLAMF6^+^ P14 cells for each infection condition, and corresponding representative overlay of SLAMF6^+^ P14 cells displayed in tSNE plots. **P < 0.01, ***P < 0.001, ****P < 0.0001. Fig. S1 shows additional supporting data. LOD, limit of detection.

While early IFNAR blockade during acute LCMV infection enhances the formation of stem-like T cells, IFN-I signaling is critical to overcome and clear viral infection (Crouse et al., 2015; Lazear et al., 2019). To determine whether d0–1 IFNAR inhibition prevented viral clearance and induced a chronic-like infection, we assessed viral load in control-treated and d0–1 IFNAR-blocked acute LCMV infection and compared these settings with chronic LCMV Docile infection. At day 8 (d8) of infection, acute LCMV–infected control mice had cleared virus, while mice that received d0–1 IFNAR block had persistent viral load (Fig. 2 A). Unlike chronic LCMV–infected mice, d0–1 IFNAR-blocked acute LCMV–infected mice cleared the infection by day 14 (d14) (Fig. 2 A). The expression of the terminal exhaustion marker TIM-3 tracked with viral load (Fig. 2 B and Fig. S1 D), and the promotion of stem-like T cells in d0–1 IFNAR-blocked mice was significantly increased compared with control-treated mice and therefore distinct from that seen in chronically infected mice (Fig. 2 C and Fig. S1 E).

Given d0–1 IFNAR blocking during acute LCMV infection resulted in the near absence of T_EFF_ cells, we further questioned whether our observation of increased T_SCM_ cell differentiation was indicative of formation of cell exhaustion. For this, we explored the differentiation of P14 cells in control and d0–1 IFNAR-blocked acute LCMV infection and chronic LCMV infection using FlowSOM dimensionality reduction analysis (Quintelier et al., 2021). We characterized five distinct populations from the concatenated d8 and d14 dataset using expression attributes of each FlowSOM-generated population (Fig. 2 D; and Fig. S1, F and G). This revealed populations 1 and 2 to be KLRG1^+^ T_EFF_ cells, distinguished by CX3CR1 expression. Population 3 had high expression of stem-like memory markers including SLAMF6, CD62L, and CD127. Population 4 was characterized as a terminally exhausted CD8^+^ T (T_EX_) cell population, with high PD-1, TIM-3, and CXCR6 expression. Finally, population 5 was determined to be effector memory CD8^+^ T cells as this population expressed high levels of CD44, CX3CR1, and SCA-1 markers. To determine how stem-like T cells compared across each experimental condition, we identified SLAMF6^+^ (as a surrogate for TCF-1 expression [Xia et al., 2022]) P14 cell populations. At d8 of acute LCMV infection, SLAMF6^+^ T cells within the control-treated and d0–1 IFNAR-blocked mice were predominantly found in population 3 (Fig. 2 E). In contrast, most SLAMF6^+^ T cells generated in the chronic LCMV infection were within population 4 (Fig. 2 E), indicating increased exhaustion. By d14 of infection, almost all SLAMF6^+^ T cells within each infection setting overlaid with population 3 with the greatest number evident in the d0–1 IFNAR-blocked mice (Fig. S1 H). Combined, stem-like T cells arising in d0–1 IFNAR-blocked acute LCMV–infected mice were distinct from those in chronic LCMV infection and remain increased following viral clearance. Thus, in comparison with previous studies that demonstrate extended IFNAR blocking induces chronicity (Teijaro et al., 2013; Wilson et al., 2013), here we show that early, short-term IFNAR inhibition delayed viral clearance but did not establish a chronic-like infection.

### T_SCM_ and T_PEX_ are transcriptionally distinct transitional cellular states

**Figure 3.**
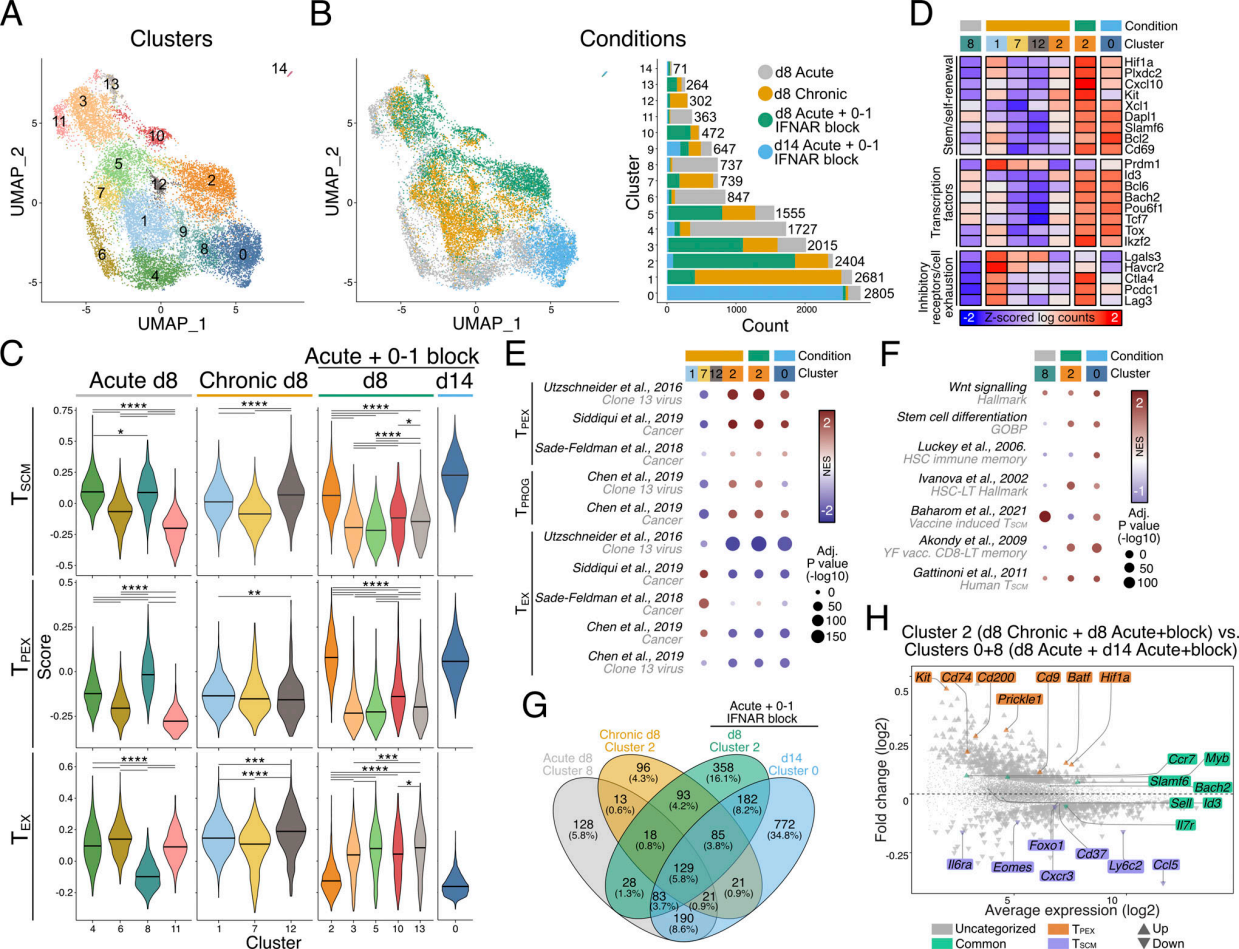
**Early IFNAR inhibition promotes transitional T**_**PEX**_ **cell formation prior to establishing the T**_**SCM**_ **cell population. (A–H)** scRNAseq analysis of 17629 P14 cells from peripheral lymph nodes at d8 acute LCMV, d8 chronic LCMV, and d8 and d14 IFNAR-blocked acute LCMV infection conditions. Data show three to four biological replicates per condition. **(A and B)** UMAPs of CD8^+^ T cells (A) based on infection condition and (B) prominence of each condition per cluster. **(C)** Module scores of T_SCM_-, T_PEX_-, and T_EX_-associated genes in prominent populations from each condition. **(D)** Normalized mean expression heat map of classed marker genes in selected clusters, normalized as z-scored log counts across conditions. **(E and F)** NES plots of enrichment of (E) T_PEX_ cell, exhausted progenitor (T_PROG_) cell, and T_EX_ cell gene programs from published datasets, and (F) enrichment of hallmark Wnt signaling and stemness signatures. **(G)** Venn diagram reflecting distinct and intersecting GEX in selected clusters. **(H)** MA plot of log fold change versus mean expression between C2 (comprising cells from chronic LCMV and d8 IFNAR-blocked acute LCMV) and C0+C8 (comprising cells from d14 IFNAR-blocked acute LCMV and control acute LCMV). Marked genes represent intersections of the Venn diagram in G, genes shared by each cluster (green), genes shared in C2 (orange), genes shared in C0+C8 (purple). DE genes identified in G came from analysis using the voom-limma pipeline with duplication correlation and P <0.05. *P < 0.05, **P < 0.01, ***P < 0.001, ****P < 0.0001. Fig. S2 shows additional supporting data. HSC-LT, long-term hematopoietic stem cells. YF, Yellow Fever.

**Figure S2.**
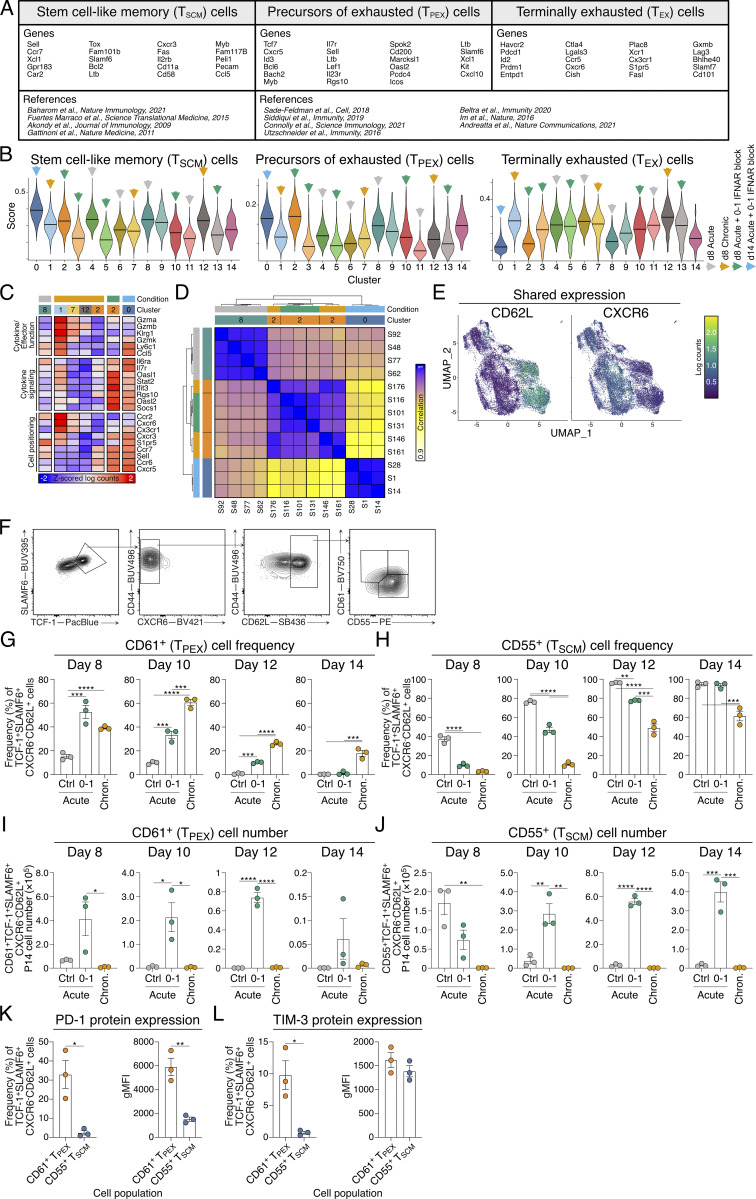
**Application of novel surface markers CD61 and CD55 to track the reversible transition of T**_**PEX**_ **to T**_**SCM**_ **cells.** Related to Figs. 3 and 4. **(A–D)** Sequencing analysis of P14 cells as described and clustered in Fig. 3 A. **(A)** Anchor genes and reference list used to define T_SCM_, T_PEX_, and T_EX_ cell states. **(B)** Module scores of T_SCM_, T_PEX_, and T_EX_ cell genes within each defined cluster. Prominent clusters in each experimental setting are indicated with arrows. Clusters without arrows indicate clusters comprised of mixed conditions (as in Fig. 3 B). **(C)** Normalized mean expression heat map of key marker genes in selected clusters. **(D)** Pearson’s correlation analysis of all genes expressed in pseudobulk samples from indicated clusters and conditions. Each row/column is an independent biological replicate. **(E)** Surface protein sequencing analysis of P14 cells as described in Fig. 3. Surface expression of CD62L and CXCR6 on UMAPs of CD8^+^ T cells. **(F–J)** Analysis of CD61 and CD55 markers on the surface of antigen-specific CD8^+^ T cells from d8 to d14 of acute LCMV with or without d0–1 IFNAR block, and chronic LCMV infection. Data are representative of three independent experiments with three mice per infection setting per time point. Each dot in G–K represents a single mouse. Data are the mean ± SEM. Statistical differences were analyzed using a one-way ANOVA test. **(F)** Gating strategy to identify TCF-1^+^SLAMF6^+^CXCR6^−^CD62L^+^CD61^+^CD55^−^ T_PEX_ cells and TCF-1^+^SLAMF6^+^CXCR6^−^CD62L^+^CD61^−^CD55^+^ T_SCM_ cells. Pre-gated on P14 cells. **(G and H)** Graphs summarizing frequencies of (G) CD61^+^ T_PEX_ and (H) CD55^+^ T_SCM_ cells throughout infection. **(I and J)** Graphs summarizing the cell numbers of (I) CD61^+^ T_PEX_ and (J) CD55^+^ T_SCM_ cells throughout infection. **(K and L)** Surface protein expression frequency (left panels) and gMFI (right panels) on CD61^+^ T_PEX_ and CD55^+^ T_SCM_ cells. Data are the mean ± SEM. Data are representative of three independent experiments with three mice per group. Statistics was determined by unpaired *t* tests. **(K)** Frequency and gMFI of PD-1 and TIM-3 on CD61^+^ T_PEX_ cells. **(L)** Frequency and gMFI of PD-1 and TIM-3 on CD55^+^ T_SCM_ cells. Data are the mean ± SEM. Data are representative of three independent experiments with three to four mice per group. *P < 0.05, **P < 0.01, ***P < 0.001, ****P < 0.0001.

TCF-1^+^SLAMF6^+^ stem-like T cells comprise both T_SCM_ cells, which are found in acute infection and vaccination, and T_PEX_ cells, which arise during chronic infection (Beltra et al., 2020; Chen et al., 2019; Utzschneider et al., 2020). The extended infection length observed with d0–1 IFNAR blocking during acute LCMV Armstrong infection provided a unique opportunity to investigate the developmental relationship between these cellular states. To this end, we performed paired single-cell RNA sequencing (scRNAseq) and surface protein sequencing (CITEseq) analysis, comparing P14 cells from d8 acute LCMV Armstrong infection (4,499 cells) and chronic LCMV Docile infection (4,967 cells) with d0–1 IFNAR-blocked LCMV Armstrong at both d8 (5,061 cells) and d14 (3,102 cells). Cluster analysis revealed 15 populations that were broadly distinct between each infectious setting (Fig. 3, A and B). To identify stem-like CD8^+^ T cell populations of interest, we used anchor genes selected from previous studies to identify the cellular states that correspond to T_SCM_, T_PEX_, and T_EX_ cells for clusters associated with each condition (Fig. 3, B and C; and Fig. S2, A and B). Due to the close relationship between T_SCM_ and T_PEX_ cells, clusters were identified with overlapping high scores for both cellular states. Nevertheless, cluster (C) 8, emerging from d8 acute LCMV infection, was identified as a naturally forming T_SCM_ cell population, having a high T_SCM_ score and a low T_EX_ score (Fig. 3 C). C2 was the major population shared between d8 d0–1 IFNAR-blocked LCMV infection and chronic LCMV infection and showed the highest score for both T_SCM_ and T_PEX_ signatures. As C2 was distinct from clusters with high T_EX_ scores (C1, 7, 12; and C3, 5, 10, 13), this suggested it represented T_PEX_ cells (Fig. 3 C). Despite falling in the same cluster, differentially regulated genes were still evident between these conditions that indicated blocking IFNAR during cell priming induced genes associated with self-renewal, longevity, and proliferation (including *Kit*, *Il7r*, *Tox*, *Bcl2*) (Alfei et al., 2019; Baharom et al., 2021) (Fig. 3 D and Fig. S2 C). Further, performing normalized enrichment score (NES) analysis with published chronic infection and cancer datasets from human and mice confirmed the identity of C2 as a T_PEX_ cell state, distinct from terminal exhaustion (Fig. 3 E).

In comparison with cell states present with active infection (chronic LCMV and d8 d0–1 IFNAR-blocked acute LCMV), C0 was the major cluster derived from d14 d0–1 IFNAR-blocked acute LCMV and had the highest T_SCM_ score of all clusters (Fig. 3, B and C). C0 expressed similar stemness genes to C2, with the decreased expression of inhibitory receptors (*Ctla4*, *Pdcd1*, *Lag3*) and increased effector molecules (*Ly6C1*, *Ccl5*), which aligned them with the naturally occurring T_SCM_ (C8) cells found in acute LCMV infection (Fig. 3 D and Fig. S2 C). NES analysis revealed that both d0–1 IFNAR-blocked C0 and C2 populations exhibited enhanced Wnt signaling and stemness signatures that were predictive of stem cell–like and long-lived vaccine responses, relative to acute LCMV memory formed without IFNAR block (Fig. 3 F). We next analyzed the intersecting genes expressed between T_PEX_ and T_SCM_ clusters to confirm 129 genes (including *Tcf7*, *Slamf6*, *Myb*, *IL7r*, *Ccr7*) that were shared genes between these closely related cellular states (Fig. 3, G and H; and Table S1). Pearson’s correlation analysis between T_PEX_ and T_SCM_ cell clusters indicated that C2 derived from chronic LCMV and acute d0–1 IFNAR-blocked LCMV was similar to each other, as were C8 and C0, comprising cells from acute LCMV and d14 acute d0–1 IFNAR-blocked LCMV, respectively (Fig. S2 D). A direct comparison of these groups indicated signature genes that were either shared (*Slamf6*, *Myb*, *Id3*, *Il7r*) or distinguished between T_PEX_ (*Hif1a*, *Batf*, *Kit*, *Cd200*) and T_SCM_ (*Eomes*, *Foxo1*, *Il6ra*, *Cxcr3*) cellular states (Fig. 3 H). The longitudinal analysis of d0–1 IFNAR-blocked LCMV P14 cells that converged into a single T_SCM_ C0 at d14 suggested that this population emerged from the earlier T_PEX_ cellular state. Importantly, these settings differ in the presence or clearance of viral load, inferring this environmental cue, rather than chronicity that precedes cellular exhaustion, discriminates T_PEX_ and T_SCM_ cell states.

### Viral load–dependent conversion from T_PEX_ to T_SCM_ cellular states

**Figure 4.**
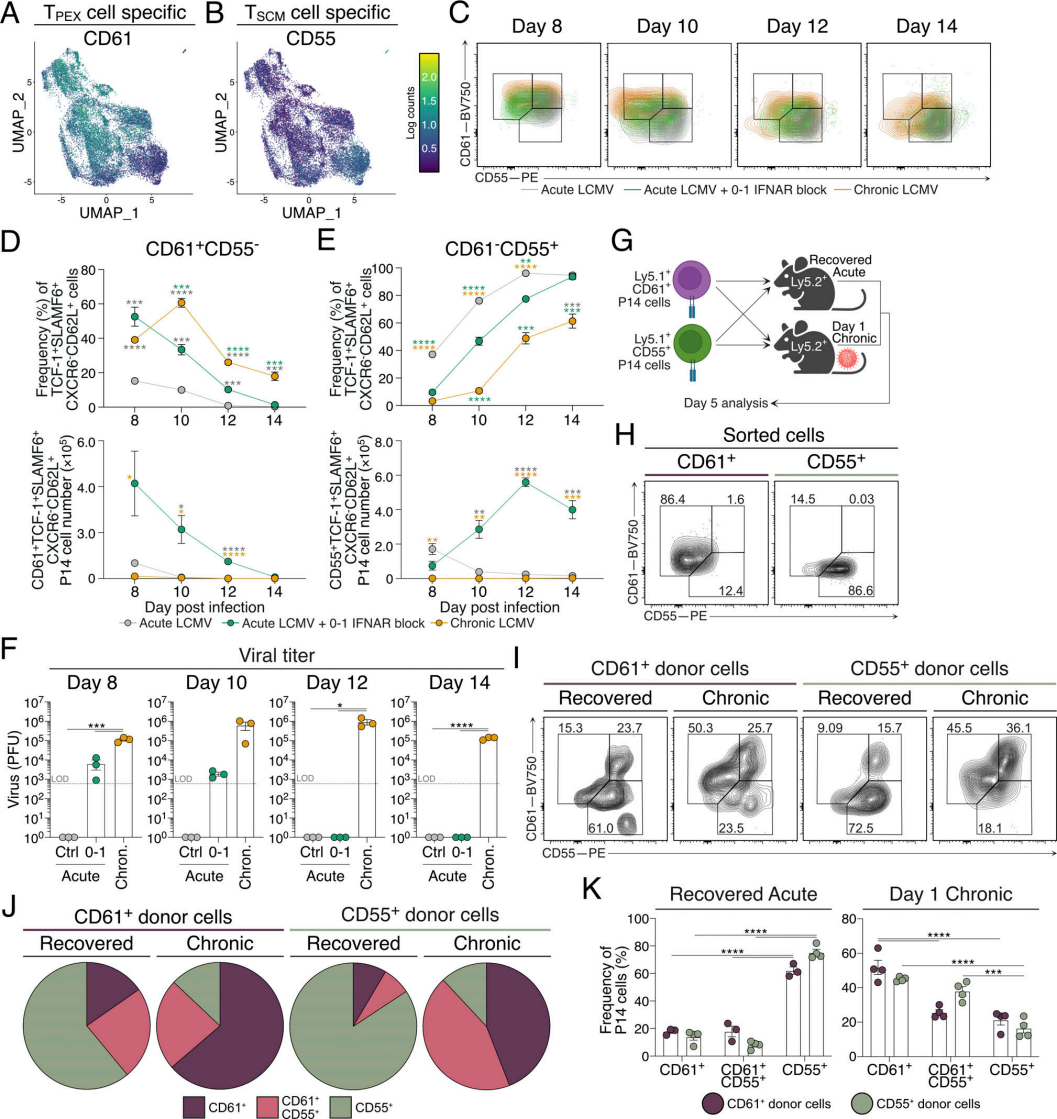
**Identification of novel cell surface markers to identify transitional stem-like CD8** ^**+**^ **T cell states that align with antigen load. (A and B)** Surface protein sequencing analysis of P14 cells as described in Fig. 3. **(A)** Surface expression of CD61 on UMAPs of CD8^+^ T cells. **(B)** Surface expression of CD55 on UMAPs of CD8^+^ T cells. **(C–E)** Flow cytometry analysis of P14 cell frequency and numbers at d8 to d14 of acute LCMV, with or without IFNAR blocking at d0–1, or chronic LCMV infection. Data are representative of two independent experiments with three mice per infection setting per time point. Data are the mean ± SEM. Statistical differences were analyzed using a one-way ANOVA test. **(C)** Overlayed representative flow cytometry plots from d8 to d14 in each experimental group. **(D and E)** Graphs summarizing the frequencies and total cell numbers of (D) CD61^+^CD55^−^ (T_PEX_) and (E) CD61^−^CD55^+^ (T_SCM_) cells throughout each infection condition. Each cell population was pre-gated on TCF-1^+^SLAMF6^+^CXCR6^−^CD62L^+^ P14 cells as shown in Fig. S2 F. **(F)** Graphs summarizing the PFU from viable virus in spleens of mice in each infection condition over the time course. The dashed line indicates viral plaque LOD. **(G–K)** Isolation, adoptive cell transfer, and analysis of CD61^+^ T_PEX_ and CD55^+^ T_SCM_ cell surface marker expression in either antigen-free or antigen-rich host mice. Data are representative of three independent experiments with three to four mice per group. Each dot in F and K represents a single mouse. Data are the mean ± SEM. Statistical differences were analyzed using a one-way ANOVA test. **(G)** Experimental scheme. CD61^+^ T_PEX_ and CD55^+^ T_SCM_ cells were sorted from d8 chronic LCMV– and day 10 acute LCMV–infected mice, respectively. Isolated cells were individually transferred into either recovered (from acute infection, antigen-free) or d1 chronically infected host mice. Mice were maintained for 5 days before subsequent cell surface marker analysis. **(H)** Flow cytometry plots analyzing the expression of CD61 and CD55 on sorted single-positive cell populations prior to adoptive cell transfer. **(I)** Representative flow cytometry plots of CD61^+^ and CD55^+^ donor T cells isolated from host mice 5 days following cell transfer. **(J)** Graphs summarizing the frequencies of CD61^+^CD55^−^ T_PEX_, CD61^+^CD55^+^, and CD61^−^CD55^+^ T_SCM_ cells shown in I. **(K)** Pie charts summarizing I and J. *P < 0.05, **P < 0.01, ***P < 0.001, ****P < 0.0001. Fig. S2 shows additional supporting data. LOD, limit of detection.

The inclusion of surface protein sequencing in our scRNAseq dataset afforded the ability to identify cell surface proteins that distinguish T_PEX_ and T_SCM_ cell clusters. Overlaying the surface protein panel onto scRNAseq Uniform Manifold Approximation and Projection (UMAP) clusters indicated that CD61 and CD55 discriminate T_PEX_ and T_SCM_ cell populations, respectively, within the greater TCF-1^+^SLAMF6^+^CXCR6^−^CD62L^+^ cell population (Fig. 4, A and B; and Fig. S2, E and F). We next examined whether these new markers of stem-like cell states enabled the delineation of conversion from T_PEX_ to T_SCM_ cells throughout the course of infection. Consistent with our scRNAseq data, P14 cells in d8 IFNAR-blocked acute LCMV and chronic LCMV infection showed a high frequency and cell number of CD61^+^ T_PEX_ cells within the TCF-1^+^SLAMF6^+^CXCR6^−^CD62L^+^ gate (Fig. 4, C and D; and Fig. S2, G and I). P14 cells in acute LCMV infection had a smaller frequency of these cells, which transitioned through a CD61^+^CD55^+^ cell population before almost entirely consisting of CD61^−^CD55^+^ T_SCM_ cells by day 12 (d12) of infection (Fig. 4, C–E and Fig. S2, G–J). While P14 cells in chronic LCMV showed some transition to CD61^−^CD55^+^ T_SCM_ cells, the CD61^+^CD55^−^ T_PEX_ cell population was maintained at d14 of infection (Fig. 4, C–E; and Fig. S2, G and H). In line with the delayed viral clearance observed with IFNAR blockade compared with control acute LCMV infection, the complete transition from CD61^+^CD55^−^ T_PEX_ to CD61^−^CD55^+^ T_SCM_ cells was delayed to d14, resulting in a significant expansion of the cells remaining following treatment (Fig. 4, C–E and Fig. S2, G–J). Consistent with the T_PEX_ cell phenotype being concomitant with viral load, conversion to the CD61^−^CD55^+^ T_SCM_ cells followed viral clearance in acute LCMV infection (Fig. 4 F). Further, as viral clearance was delayed during d0–1 IFNAR-blocked acute LCMV infection, with clearance at d12 rather than d8 for control infection, this aligned with the delayed transition of T_PEX_ to T_SCM_ cellular states (Fig. 4 F). This suggests that the presence of antigen load discriminates these two states, and that once cleared, T_PEX_ cells transition into T_SCM_ cells to maintain the memory T cell pool.

### Stem-like CD8^+^ T cell transition is plastic and instructed by antigen presence

The observation that T_PEX_ cells transition into T_SCM_ cells following viral clearance raised questions regarding the plasticity of these cellular states; that is, whether cells can revert from an established T_SCM_ memory cell phenotype when reexposed to antigen. To investigate this, we sorted congenically labeled CD61^+^ T_PEX_ cells and CD55^+^ T_SCM_ cells from chronic and acute infected mice, respectively (Fig. 4, G and H). Each purified cell state was then transferred into either mice that had recovered from acute LCMV infection, or day 1 (d1) chronically infected mice. 5 days following transfer, irrespective of their sorted phenotype, stem-like CD8^+^ T cells gave rise to cellular states instructed by the presence or absence of virus, such that when transferred into recovered mice, CD55^+^ expression was either maintained (for CD55^+^ T_SCM_ sorted cells) or increased (for CD61^+^ T_PEX_ sorted cells) (Fig. 4, I–K). Similarly, cells transferred into chronically infected mice shared features of CD61^+^ T_PEX_ cells regardless of their sorted status 5 days prior (Fig. 4, I–K). Conversion from CD61^+^ T_PEX_ to CD55^+^ T_SCM_ cells was associated with PD-1 and TIM3, corresponding to regulation of inhibitory receptors observed in our scRNAseq experiments (Fig. 3 D; and Fig. S2, K and L). We noted some transferred cells in each setting were CD61^+^CD55^+^, likely representing cells undergoing active transition (Fig. 4, I–K). Thus, T_PEX_ and T_SCM_ cells interconvert based on the antigenic environment, indicating a reversible developmental trajectory between these cellular states.

### Early IFNAR blocking increases the expression of dendritic cell (DC)–derived CXCR3 ligands

**Figure 5.**
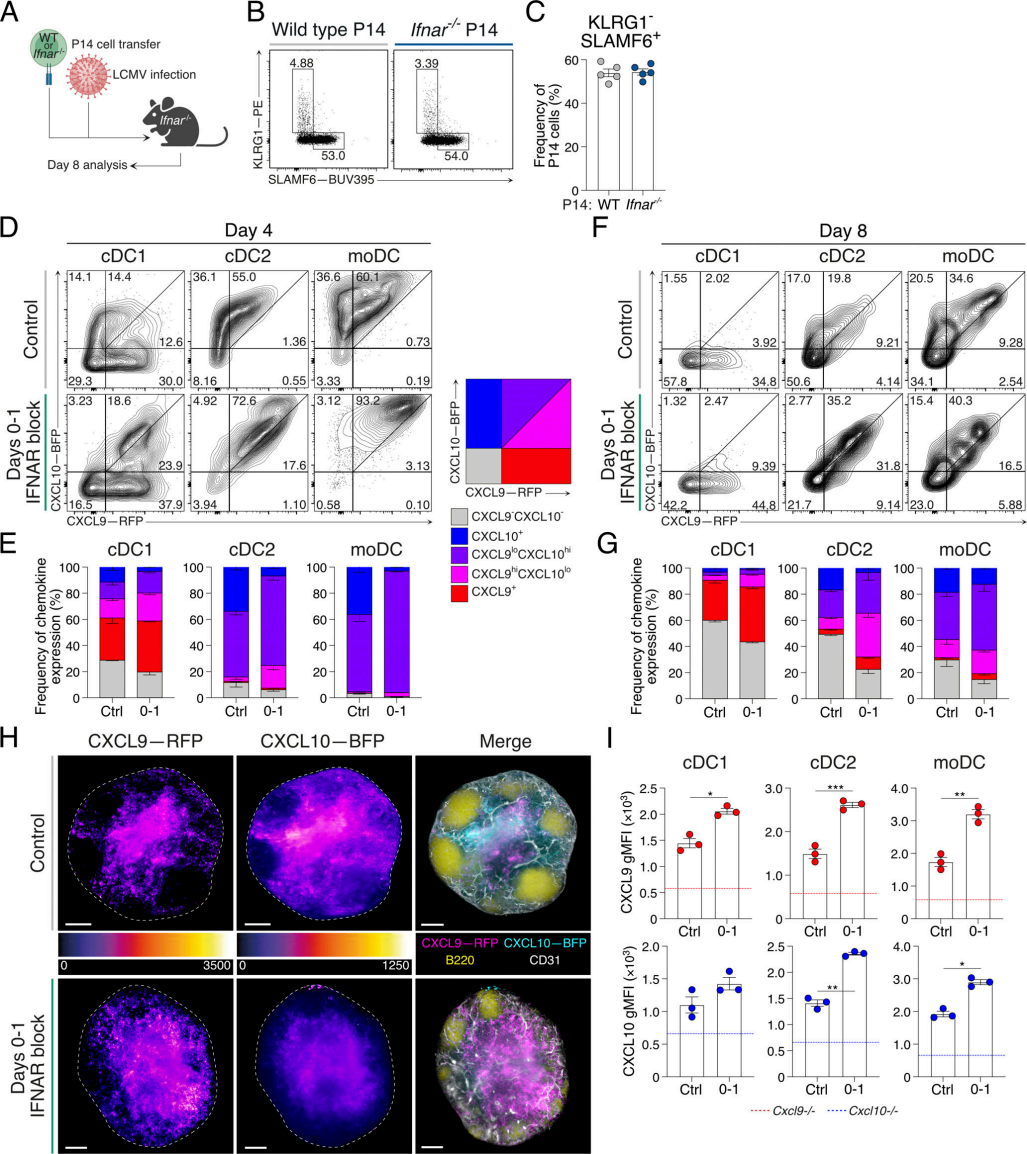
**IFNAR blocking increases DC expression of CXCR3 ligands following immune challenge. (A–C)** Analysis of intrinsic IFNAR signaling on the differentiation of CD8^+^ T cells. Data are representative of two individual experiments with five mice in each group. Each dot in C represents a single mouse. **(A)** Experimental scheme. Wild-type or *Ifnar*^*−/−*^ P14 cells were transferred into *Ifnar*^*−/−*^ host mice prior to acute LCMV infection and analysis at d8. **(B)** Representative flow cytometry plots. **(C)** Average frequency ± SEM of T_SCM_ cell populations. **(D–G)** REX3 reporter expression in cDC1, cDC2, and moDC populations during acute LCMV infection in control and d0–1 IFNAR-blocked mice. Data are representative of three individual experiments with three to four mice in each group in each experiment. Data are the mean ± SEM. **(D and F)** (D) d4 and (F) d8 post infection representative plots showing *Cxcl9*-RFP and *Cxcl10*-BFP reporter expression in indicated DC subsets. **(E and G)** Graphs summarizing graded frequencies of *Cxcl9*-RFP and *Cxcl10*-BFP expression from D and F, respectively. Graded expression summarized in key. **(H)** LSFM micrographs of intact REX3 lymph nodes at d8 of acute LCMV infection in control and d0–1 IFNAR-blocked conditions. Images are 200-μm longitudinal slices through the lymph node center. Scale bars represent 100 μm. The dashed line indicates the lymph node outline. Pseudocolor FIRE LUT heat maps for each REX3 reporter (left and middle panels). Merged images (right panels) show *Cxcl9*-RFP (magenta), *Cxcl10*-BFP (cyan), B220 (B cells; yellow), and CD31 (vessels; white). Images are representative of two individual experiments with four mice in each group in each experiment. **(I)** Detection of CXCL9 and CXCL10 protein staining in cDC1, cDC2, and moDC populations at d4 of acute LCMV infection. Graphs show average gMFI ± SEM. The dashed line indicates chemokine protein detection in indicated DC subsets from *Cxcl9*^*−/−*^ and *Cxcl10*^*−/−*^ mice. Data are representative of three individual experiments with at least three mice in each group in each experiment. Each dot represents a single mouse. Statistics was determined using unpaired *t* tests. *P < 0.05, **P < 0.01, ***P < 0.001. Fig. S3 shows additional supporting data.

**Figure S3.**
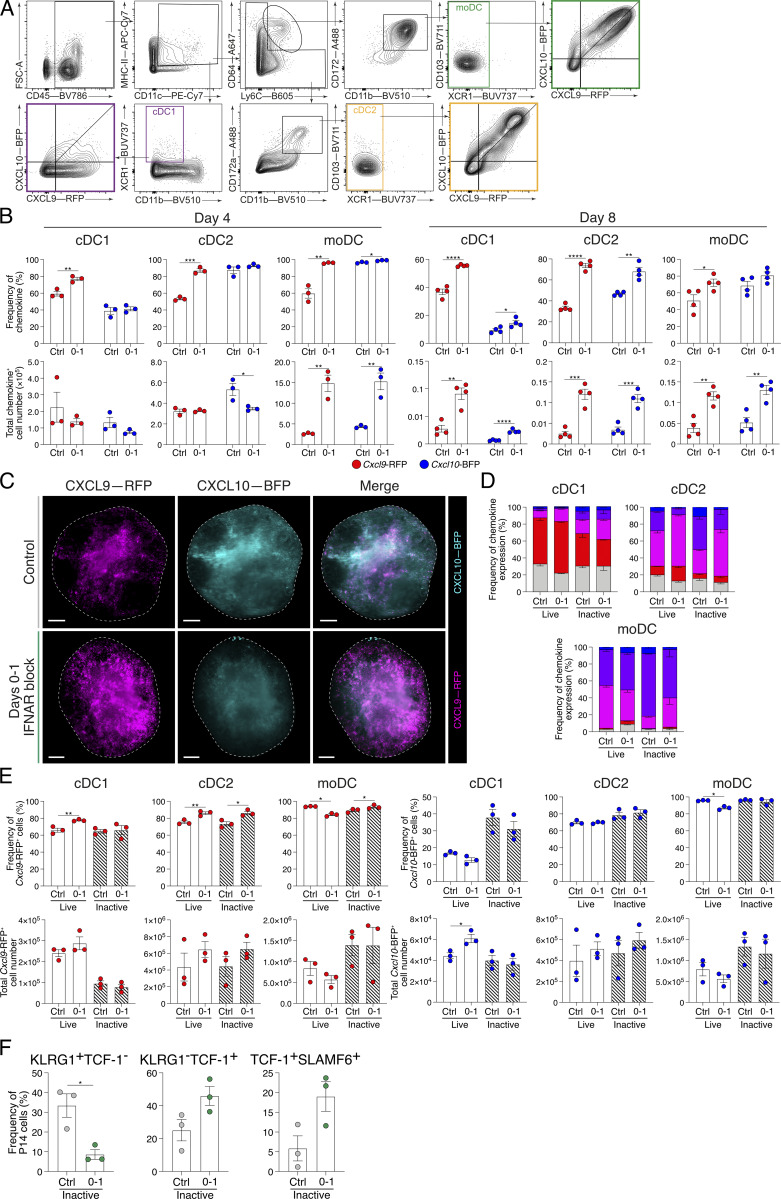
**DC gating strategy and increased CXCR3 ligand expression and T**_**SCM**_ **cell differentiation during live and heat-inactivated acute LCMV infection following IFNAR blocking.** Related to Fig. 5. **(A)** Gating strategy to identify cDC1, cDC2, and moDC cell populations. Colored boxes indicate the final gate and REX3 expression for each subset. **(B)** Frequency and total cell number of *Cxcl9*-RFP^+^ and *Cxcl10*-BFP^+^ DC subsets at d4 and d8 after LCMV Armstrong infection. Data are representative of three individual experiments with three to four mice in each group. Data are the mean ± SEM. Each dot represents a single sample. Statistical differences were analyzed using unpaired *t* tests. **(C)** Images from Fig. 5 H. LSFM micrographs of intact REX3 lymph nodes at d8 of infection in control and d0–1 IFNAR-blocked conditions. Images are 200-µm longitudinal slices through the lymph node center. Scale bars represent 100 µm. The dashed line indicates the lymph node outline. Individual REX3 reporters and merge images show *Cxcl9*-RFP (magenta) and *Cxc10*-BFP (cyan). Images are representative of three individual experiments with at least four mice in each group in each experiment. **(D and E)** Analysis of *Cxcl9*-RFP and *Cxcl10*-BFP expression in DC subsets within peripheral lymph nodes of mice at d8 following challenge with either live or heat-inactivated (inactive) LCMV Armstrong. Half of each group received d0–1 treatments of IFNAR block. Data are representative of three individual experiments with three mice per group per experiment. Data are the mean ± SEM. **(D)** Graphs summarizing the graded frequencies of *Cxcl9*-RFP^+^ and *Cxcl10*-BFP^+^ DC subsets at d8 following acute LCMV Armstrong infection. **(E)** Frequency and total cell numbers and gMFI of chemokine reporter expression in each immune challenge condition. Each dot represents a single mouse. Data are the mean ± SEM. Statistical differences were analyzed using unpaired *t* tests. **(F)** Graphs summarizing the mean frequencies ± SEM of T_EFF_ and T_SCM_ cells in each condition. Data are representative of three independent experiments with three to four mice in each group. Statistical differences were analyzed using unpaired *t* tests. *P < 0.05, **P < 0.01, ***P < 0.001, ****P < 0.0001.

IFN-I can influence T cell differentiation, by both cell-intrinsic and cell-extrinsic mechanisms (Bullock et al., 2024; Crouse et al., 2015; Huang et al., 2019; Lazear et al., 2019). To untangle the direct requirement for IFNAR inhibition, we transferred wild-type or *Ifnar*^*−/−*^ P14 cells into *Ifnar*^*−/−*^ hosts prior to acute LCMV infection (Fig. 5 A). The lack of significance between the frequency of T_SCM_ cells formed by wild-type and *Ifnar*^*−/−*^ P14 cells in *Ifnar*^*−/−*^ hosts (Fig. 5, B and C) indicated that IFNAR blocking primarily modulated the T_SCM_ cell fate via altering the T cell microenvironment, rather than via T cell–intrinsic mechanisms. We therefore sought to determine the underlying microenvironmental mechanisms of IFNAR blockade in driving T_SCM_ cell differentiation. We reasoned this could be due to decreased inflammation and/or via disruption of chemokine gradients that would otherwise promote T_EFF_ cell differentiation (Duckworth et al., 2021). The expression of CXCR3 ligands, CXCL9 and CXCL10, is key IFN-induced environmental cues that regulate T cell positioning and function during acute and chronic viral infection, and recall responses (De Giovanni et al., 2020; Duckworth et al., 2021; Ozga et al., 2022; Sung et al., 2012). We predicted that d0–1 IFNAR blockade would reduce their expression, resulting in cell retention in the lymph node paracortex to favor T_SCM_ cell differentiation (Duckworth et al., 2021). To examine this question, we used the dual reporter of CXCR3 ligands, the REX3-transgenic mouse, to identify the DC chemokine cellular sources (Groom et al., 2012) (Fig. S3 A). In contrast to our prediction, on d4 of acute LCMV infection, the frequency and the total number of *Cxcl9*-RFP– and *Cxcl10*-BFP–expressing cells were increased in d0–1 IFNAR-blocked REX3 mice, compared with the control (Fig. 5, D and E; and Fig. S3 B). The frequency of *Cxcl9*-RFP expression was increased in conventional types 1 and 2 dendritic cells (cDC1 and cDC2, respectively), while the expression of both *Cxcl9* and *Cxcl10* was increased in inflammatory monocyte–derived DCs (moDCs) (Fig. S3 B). Increased chemokine expression continued to be observed in d0–1 IFNAR-blocked REX3 mice at d8 of acute LCMV infection (Fig. 5, F and G; and Fig. S3 B). This was confirmed by light-sheet fluorescence microscopy (LSFM) of whole, cleared lymph nodes at d8 following acute LCMV infection. Scaled pseudocolor images indicated increased *Cxcl9*-RFP chemokine abundance and disrupted *Cxcl10*-BFP gradient formation in d0–1 IFNAR-blocked lymph nodes (Fig. 5 H, Fig. S3 C, and Video 1).

**Video 1.** **Increased CXCR3 chemokine abundance with early IFNAR blockade.** Related to Fig. 5. Expression of *Cxcl9*-RFP and *Cxcl10*-BFP reporters in control or d0–1 αIFNAR-treated mice (as in Fig. 5 H). Representative LSFM 3D images of cleared intact inguinal lymph nodes in control (left) or d0–1 IFNAR-blocked (right) mice showing *Cxcl9*-RFP (magenta), *Cxcl10*-BFP (cyan), B220 (B cells; yellow), and CD31 (vessels; white).

As REX3 mice report the expression of *Cxcl9* and *Cxcl10* mRNA, it was important to confirm that chemokine expression was increased at the protein level following d0–1 IFNAR inhibition. For this, chemokine secretion was blocked 4 h prior to harvest at d4 of acute LCMV infection. In line with the increased REX3 reporter expression, CXCL9 and CXCL10 showed increased gMFI following d0–1 IFNAR block, compared with control-treated cDC1, cDC2, and moDC populations (Fig. 5 I). Therefore, d0–1 IFNAR blockade induced a paradoxical upregulation of IFN-induced chemokines during acute LCMV infection.

To determine whether the increase in CXCR3 chemokine expression following d0–1 IFNAR blocking was driven by the active viral replication and high viral load, REX3-transgenic mice were inoculated with either live or heat-inactivated replication-deficient acute LCMV. Half of each challenged cohorts were treated at d0–1 of the immune challenge, and DC populations were analyzed at d8 after infection. An increase in CXCR3 ligand expression, primarily *Cxcl9*-RFP reporter expression in the d0–1 IFNAR-blocked group, was observed irrespective of active or inactivated virus (Fig. S3, D and E). These results align with decreased T_EFF_ and increased T_SCM_ cell formation with d0–1 IFNAR blockade alongside heat-inactivated acute LCMV challenge (Fig. S3 F). Combined, these data indicate that d0–1 IFNAR inhibition promotes abundant CXCL9 and CXCL10 expression by cDC1, cDC2, and moDC populations, even in the absence of active viral replication, and this correlates with increased T_SCM_ cell formation.

### Early IFNAR inhibition leads to CD8^+^ T cell retention within the lymph node paracortex and is associated with CXCR3 desensitization

**Figure 6.**
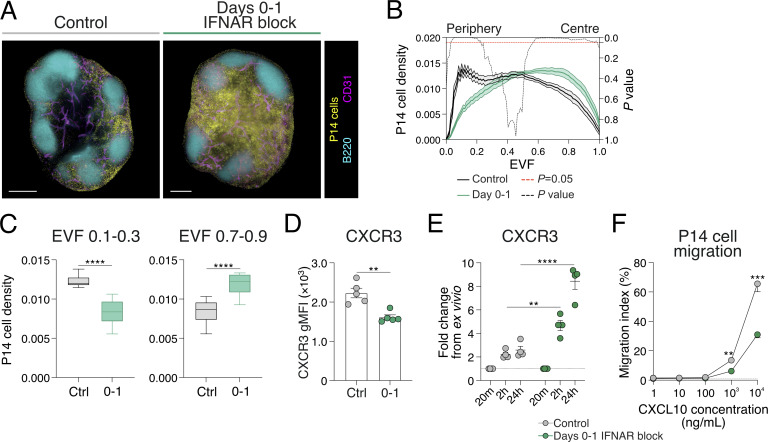
**CD8** ^**+**^ **T cell retention in the lymph node center is associated with CXCR3 desensitization. (A–C)** P14 cell positioning in lymph nodes at d8 of acute LCMV infection in control and d0–1 IFNAR-blocked mice. Images and graphs are representative of three individual experiments with four to five mice in each group per experiment. **(A)** LSFM micrographs of intact lymph nodes. Images are 200-µm longitudinal slices through the lymph node center. Scale bars represent 200 µm. Images show GFP-P14 cells (yellow), B220 (B cells; cyan), and CD31 (vessels; magenta). **(B)** Graph summarizing density of P14 cells within the 3D lymph node, from periphery (EVF = 0) to center (EVF = 1). The black dashed line indicates multiple *t* tests between P14 cell density in the two treatment conditions for each EVF value. The red dashed line indicates P = 0.05. Data are the mean ± SEM. **(C)** Graphs summarizing average density ± SEM of P14 cells within indicated regions, IFRs (EVF 0.1–0.3), and T cell paracortex (EVF 0.7–0.9). **(D–F)** Analysis of P14 cells at d8 of acute LCMV infection in control and d0–1 IFNAR-blocked mice. Data are representative of two independent experiments with four to five mice per group in each experiment. Each dot in D and E represents a single mouse. Data are the mean ± SEM. Statistical differences were analyzed using one-way ANOVA. **(D)** Graph summarizing the gMFI of P14 cell CXCR3 expression. **(E)** Graph summarizing the upregulation of CXCR3 on P14 cell surface at different time points following cell isolation. **(F)** Migratory capacity of P14 cells at different concentrations of CXCL10. **P < 0.01, ***P < 0.001, ****P < 0.0001.

T_SCM_ cell differentiation is associated with retention in the T cell paracortex in IFNAR-deficient hosts (Duckworth et al., 2021). This appeared at odds with the observation that d0–1 IFNAR blocking treatment increased CXCR3 chemokine expression (Fig. 5). To assess how T cell location was altered with early, transient IFNAR blockade, green fluorescent protein (GFP)–labeled P14 cells were transferred into mice prior to acute LCMV infection and control or d0–1 IFNAR blocking treatments. On d8 of infection, popliteal lymph nodes were cleared and analyzed by LSFM. As expected, in control-treated mice, GFP-P14 cells displayed a bimodal distribution of cells, where the majority of P14 cells were positioned surrounding the B cell follicles and deep in the IFRs, while fewer cells remained in the lymph node paracortex (Fig. 6 A and Video 2). In d0–1 IFNAR-blocked lymph nodes, the noted increase in P14 cell proliferation was evident (Fig. 1, D and E; and Fig. 6 A). Due to increased cell number, GFP-P14 cells were broadly distributed throughout the lymph node. However, compared with control-treated cells, P14 cell location in d0–1 IFNAR-blocked lymph nodes was biased toward the lymph node paracortex, consistent with previous observations in *Ifnar*^*−/−*^ mice (Duckworth et al., 2021). To quantify three-dimensional (3D) T cell location, we used eroded volume fraction (EVF) analysis to evenly divide the lymph nodes and account for differences in tissue size (Duckworth et al., 2021). In control-treated lymph nodes, GFP-P14 cells positioned toward the lymph node periphery. In contrast, GFP-P14 cells in d0–1 IFNAR-blocked lymph nodes were more homogeneously distributed with a shift toward the lymph node center (Fig. 6 B). Investigating the IFRs (EVF 0.1–0.3) and paracortex (EVF 0.7–0.9) in detail confirmed the central location of P14 cells in d0–1 IFNAR block lymph nodes (Fig. 6 C). Therefore, despite increased chemokine expression, antigen-specific CD8^+^ T cells in d0–1 IFNAR-blocked mice are retained in the lymph node paracortex, consistent with imprinting the T_SCM_ cell fate in this location.

**Video 2.** **Early IFNAR inhibition leads to CD8** ^**+**^ **T cell retention within the lymph node paracortex.** Related to Fig. 6. GFP-P14 cells in LCMV-infected control or d0–1 IFNAR-treated mice (as in Fig. 6 A). Representative LSFM 3D images of cleared intact inguinal lymph nodes in control-treated (left) or d0–1 IFNAR-blocked (right) mice showing GFP-P14 cells (yellow), B220 (B cells; cyan), and CD31 (vessels; magenta).

The paracortex positioning with abundant chemokine expression following d0–1 IFNAR blockade suggested cell retention may be mediated via CXCR3 receptor desensitization. This phenomenon is well established in in vitro migration assays where high chemokine concentration results in G protein uncoupling, receptor internalization, and cell stasis (Lammermann and Kastenmuller, 2019). However, how this process may control in vivo cell migration and positioning during inflammation is unknown. To investigate this, we compared the cell surface expression of CXCR3 on P14 cells in control-treated and d0–1 IFNAR-blocked mice infected with acute LCMV. We found CXCR3 staining was decreased in mice that received d0–1 IFNAR blocking, consistent with them residing in a high chemokine concentration environment (Fig. 6 D). This was due to, at least in part, receptor internalization, as resting cells increased surface CXCR3 detection relative to control-treated cells (Fig. 6 E). Consistent with this, P14 cells from d0–1 IFNAR-blocked mice had reduced capacity to migrate toward CXCL10 in ex vivo transwell migration assays (Fig. 6 F). Overall, these results suggest that the overexpression of chemokine following d0–1 IFNAR inhibition reduces CXCR3-directed migration to promote CD8^+^ T cell retention within the lymph node paracortex where the T_SCM_ cell fate is imprinted.

### IFNAR blockade increases IFNγ production to tune CXCR3 chemokine expression

**Figure 7.**
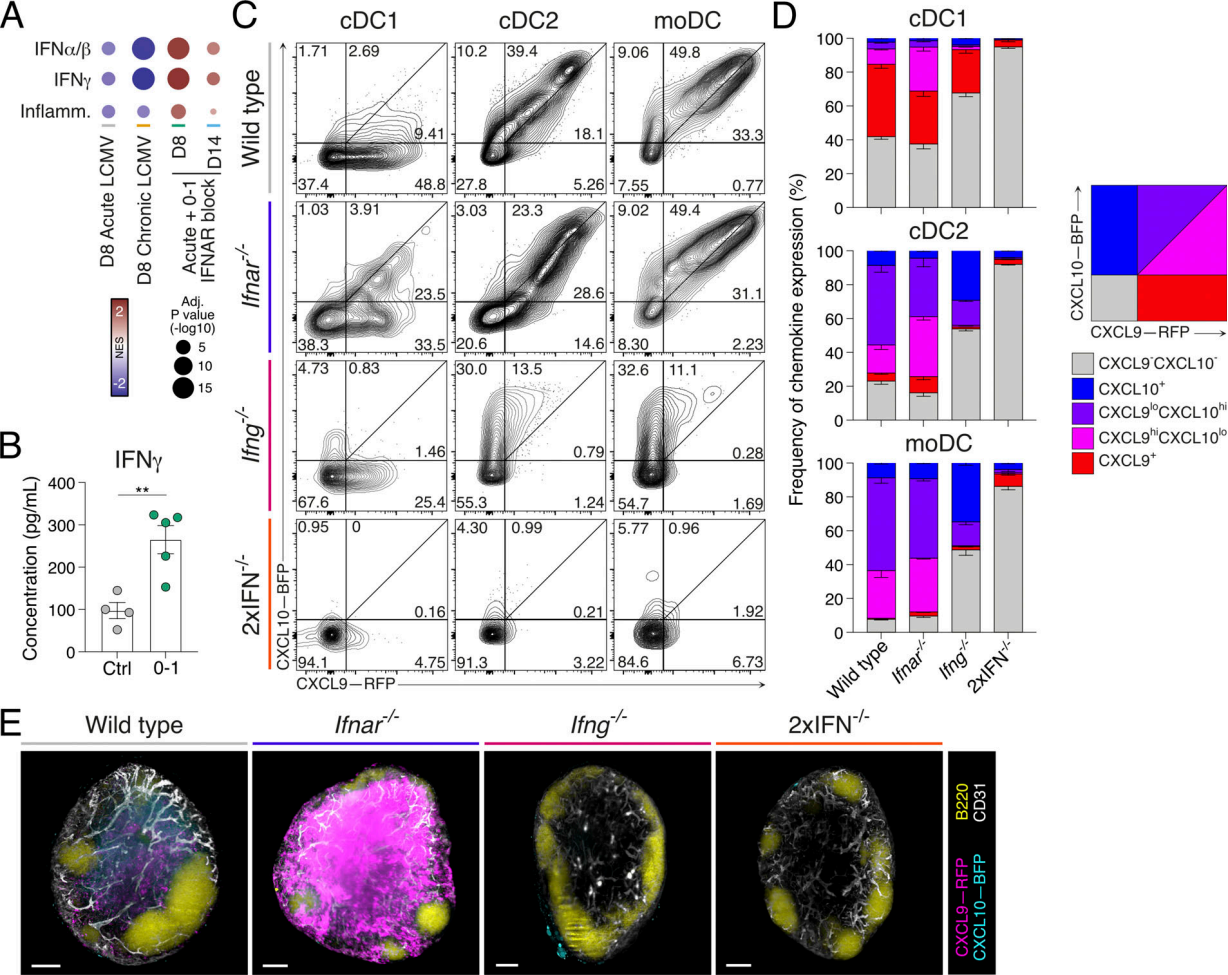
**Compensatory IFNγ drives increased CXCR3 ligands in the absence of IFNAR signaling. (A)** NES plot of hallmark type I and II interferon, and inflammation responses. Data are generated as indicated in Fig. 3. NES represents the fold change of all cells analyzed in each condition, relative to expression in all other conditions. **(B)** Concentration of IFNγ in lysates of lymph nodes at d8 following acute LCMV infection. Each dot represents a single mouse sample. Data are the mean ± SEM. Statistical differences were analyzed using one-way ANOVA tests. **(C and D)** Expression of *Cxcl9*-RFP and *Cxcl10*-BFP in cDC1, cDC2, and moDC populations from REX3 hosts crossed to indicated IFN-deficient mice at d8 of acute LCMV infection. 2xIFN^−/−^ = *Ifnar*^*−/−*^*Ifng*^*−/−*^. Data are representative of three individual repeats with at least three mice in each group in each experiment. **(C)** Representative flow cytometry plots. **(D)** Graphs summarizing graded frequencies of *Cxcl9*-RFP and *Cxcl10*-BFP expression from C. Graded expression summarized in key. **(E)** LSFM micrographs of intact wild-type, *Ifnar*^*−/−*^, *Ifng*^*−/−*^, or 2xIFN^−/−^ mouse REX3 lymph nodes. Images are 200-µm longitudinal slices through the lymph node center. Scale bars represent 100 µm. Images show *Cxcl9*-RFP (magenta), *Cxcl10*-BFP (cyan), B220 (B cells; yellow), and CD31 (vessels; white). Images are representative of three individual experiments with at least three mice in each group in each experiment. **P < 0.01. Fig. S4 shows additional supporting data.

**Figure S4.**
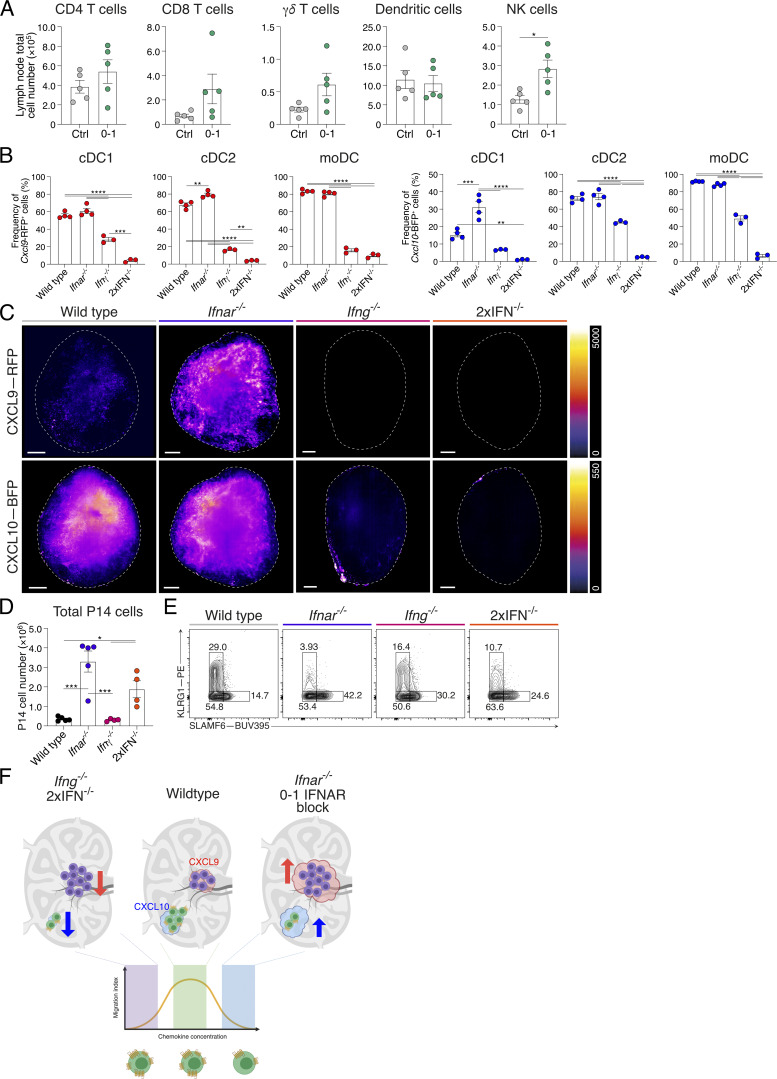
**Absence of IFN-I and IFN-II signaling promotes T**_**SCM**_ **cell differentiation during acute LCMV Armstrong infection.** Related to Figs. 7 and 8. **(A)** Total lymph node IFNγ^+^ of indicated cells from control-treated and d0–1 IFNAR-blocked mice at d4 of acute LCMV Armstrong infection. **(B)** Expression of *Cxcl9*-RFP and *Cxcl10*-BFP in cDC1, cDC2, and moDC populations from REX3 hosts crossed to indicated IFN-deficient mice. **(C)** Images from Fig. 7 E. LSFM micrographs of intact wild-type, *Ifnar*^*−/−*^, *Ifng*^*−/−*^, and 2xIFN^−/−^ mouse REX3 lymph nodes. Images are 200-µm longitudinal slices through the lymph node center. Scale bars represent 100 µm. The dashed line indicates the lymph node outline. Pseudocolor FIRE LUT heat maps for each REX3 reporter. Images are representative of three individual experiments with at least three mice in each group in each experiment. **(D)** Total P14 cell number in each wild-type or IFN-deficient host mouse. **(E)** Representative plots of P14 cells showing T_EFF_ (KLRG1^+^SLAMF6^−^), T_SCM_ (KLRG1^−^SLAMF6^+^), and T_EX_ (KLRG1^−^SLAMF6^−^) cells. **(F)** Model of IFN control of chemokine production and CD8^+^ T cell position within lymph nodes and how this correlates with in vitro migration assay chemokine concentration. Lymph nodes indicate P14 cell location and chemokine receptor expression in *Ifng*^*−/−*^ and 2xIFN^−/−^ (left), wild-type (middle), and *Ifnar*^*−/−*^ and d0–1 IFNAR-blocked (right) settings. Dotted lines indicate correlation to chemokine concentration and the bell curve of the cell migration index in in vitro assays. The wild-type setting correlates with optimal CXCL9 (red) and CXCL10 (blue) gradient formation to facilitate the generation of both T_EFF_ (green) and T_SCM_ (purple) cells. *Ifng*^*−/−*^ and 2xIFN^−/−^ settings exhibit low CXCL9 and CXCL10 expression to promote cell retention in the paracortex, increasing the generation of T_SCM_ cells and reducing T_EFF_ cells. In *Ifnar*^*−/−*^ and d0–1 IFNAR-blocked settings, increased CXCL9 and CXCL10 expression causes downregulation of surface CXCR3, preventing cell migration and increased T_SCM_ cell and reduced T_EFF_ cell differentiation. Each dot in A, B, and D represents a single sample. *P < 0.05, **P < 0.01, ***P < 0.001, ****P < 0.0001.

The upregulation of CXCR3 chemokines with IFNAR inhibition was in stark contrast to the expected regulation of these ligands (Groom and Luster, 2011a, 2011b) and suggested that d0–1 IFNAR treatment may lead to an increase in an alternative IFN pathway. NES analysis of P14 scRNAseq data from control or IFNAR-blocked acute LCMV infection indicated enhanced hallmark signatures of inflammation, IFN-I and IFN-II signaling in IFNAR-blocked samples. This highlighted the considerable overlap in IFN-I– and IFN-II–induced signatures and unappreciated compensation between these IFN families (Fig. 7 A) (Lazear et al., 2019; Rusinova et al., 2013; Walker et al., 2021). Indeed, analysis of lymph node protein lysates showed the increased expression of IFNγ in acute LCMV–infected d0–1 IFNAR-blocked mice (Fig. 7 B), with IFNγ^+^ natural killer (NK) cell numbers being significantly increased (Fig. S4 A). Therefore, we next crossed REX3 reporter mice to generate chemokine reporters deficient in IFNAR (*Ifnar*^*−/−*^), which phenotypically align with IFNAR antibody blockade (Hwang et al., 1995; Sheehan et al., 2006), IFNγ (*Ifng*^*−/−*^), or both (referred to as 2xIFN^−/−^ hereafter). This allowed us to investigate how deficiency in both IFN-I and IFN-II pathways impacted the expression of *Cxcl9*-RFP and *Cxcl10*-BFP following acute LCMV infection. Consistent with d0–1 IFNAR blocking (Fig. 5), CXCR3 chemokine expression in *Ifnar*^*−/−*^ lymph nodes was elevated in terms of the frequency of *Cxcl9*-RFP– and *Cxcl10*-BFP–expressing DCs (Fig. 7, C and D; and Fig. S4 B). In contrast, the expression of *Cxcl9*-RFP and *Cxcl10*-BFP was decreased in *Ifng*^*−/−*^ (Fig. 7, C and D; and Fig. S4 B), with near total loss of *Cxcl9*-RFP expression. In 2xIFN^−/−^ mice, chemokine expression was largely absent. Popliteal lymph nodes from each IFN genotype were cleared and analyzed by LSFM. Again, we used scaled pseudocolor maps of individual REX3 reporter proteins to observe the relative intensity of expression in each IFN genotype. REX3 chemokine reporter expression in *Ifnar*^*−/−*^ mice showed a striking upregulation of *Cxcl9*, with *Cxcl10* also being significantly upregulated (Fig. 7 E and Fig. S4 C). Similar to expression profiles shown by flow cytometry analysis, *Ifng*^*−/−*^ and 2xIFN^−/−^ mice showed a marked loss of both *Cxcl9* and *Cxcl10* (Fig. 7 E and Fig. S4 C). In addition to DC sources, REX3 chemokines are expressed by the lymph node stromal cell compartment (Alexandre et al., 2022; Duckworth et al., 2021; Groom et al., 2012; Rodda et al., 2018). As altered REX3 expression was more pronounced between the quantified DC subsets and imaged intact lymph nodes (Fig. 7, C–E), we suggest a conserved IFNγ-dominant regulation of chemokine expression governs both DC and stromal cell sources. Thus, rather than the expected diminishing of inflammatory signals, early IFN-I blockade increases the expression of other inflammatory mediators, in particular IFNγ.

### T_SCM_ cell formation is coupled to retention within the T cell paracortex

**Figure 8.**
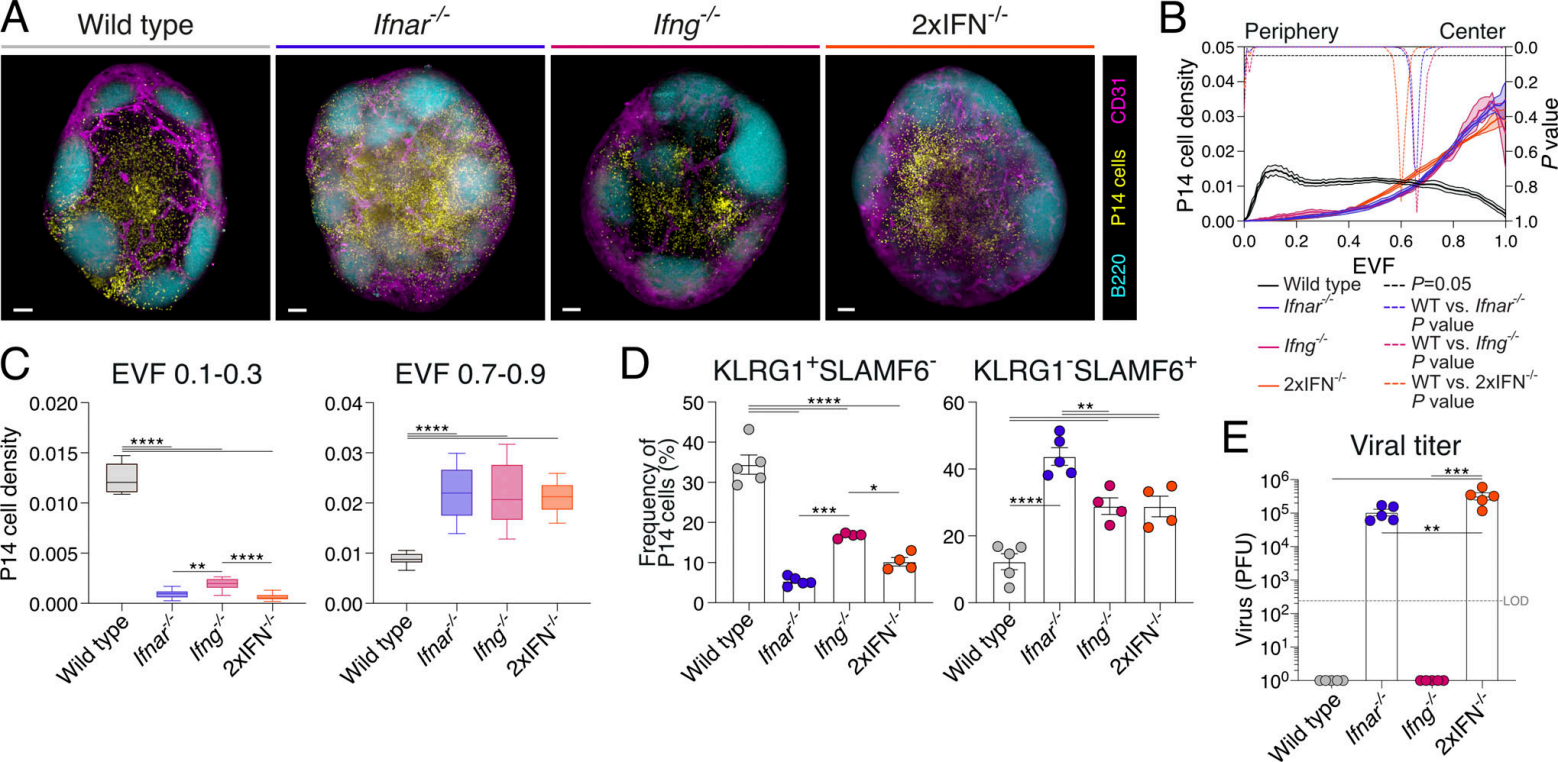
**Interplay of type I and II interferons promotes T**_**SCM**_ **cell differentiation via multiple mechanistic pathways. (A–C)** P14 cell positioning at d8 of acute LCMV infection in wild-type, *Ifnar*^*−/−*^, *Ifng*^*−/−*^, or 2xIFN^−/−^ mice. Images and graphs are representative of three individual experiments with three to five mice in each group per experiment. **(A)** LSFM micrographs of intact lymph nodes. Images are 200-µm longitudinal slices through the lymph node center. Scale bars represent 200 µm. Images show GFP-P14 cells (yellow), B220 (B cells; cyan), and CD31 (vessels; magenta). **(B)** Graph summarizing density of P14 cells within the 3D lymph node, from periphery (EVF = 0) to center (EVF = 1). The colored dashed line indicates multiple *t* tests between P14 cell density in the three experimental conditions against the wild type for each EVF value. The black dashed line indicates P = 0.05. Data are the mean ± SEM. **(C)** Graphs summarizing density of P14 cells within indicated regions, IFRs (EVF 0.1–0.3) and T cell paracortex (EVF 0.7–0.9). Data are the mean ± SEM. Statistical differences were analyzed using an unpaired *t* test. **(D)** Graphs summarizing T_EFF_ (KLRG1^+^SLAMF6^−^) and T_SCM_ (KLRG1^−^SLAMF6^+^) cell populations in each of the four conditions. **(E)** Graph summarizing the PFU from viable virus in spleens of mice in each genotype. The dashed line indicates viral plaque LOD. Each dot in D and E represents a single mouse. Data are the mean ± SEM. Statistical differences were analyzed using one-way ANOVA tests. *P < 0.05, **P < 0.01, ***P < 0.001, ****P < 0.0001. Fig. S4 shows additional supporting data. LOD, limit of detection.

Considering the disparity in CXCR3 chemokine expression with deficiency of IFNAR and/or IFNγ, we next sought to understand the impact of this on CD8^+^ T cell location. The location of GFP-labeled P14 cells was assessed in wild-type and IFN-deficient lymph nodes at d4 following acute LCMV infection. In control-treated mice, GFP-P14 cells again primarily distributed in the IFRs, while fewer remained in the lymph node center (Fig. 8 A). Quantification of P14 cell density throughout the lymph node, within the periphery (EVF 0.1–0.3) and paracortex (EVF 0.7–0.9), again highlighted a high proportion of T cells toward the outer niches of the lymph node (Fig. 8, B and C). In contrast, analysis of GFP-P14 cell distribution within *Ifnar*^*−/−*^, *Ifng*^*−/−*^, and 2xIFN^−/−^ lymph nodes revealed sequestering of T cells within the central paracortex region (Fig. 8, A–C). Additionally, the increased differentiation of T_EFF_ cells in *Ifng*^−/−^ mice was observed (Fig. 8 D and Fig. S4 E), with significantly more cells moving into the IFRs of the lymph node in this setting compared with either *Ifnar*^−/−^ or 2xIFN^−/−^ hosts (Fig. 8, B and C). As seen with d0–1 IFNAR blocking, IFNAR deficiency, either alone or in combination with *Ifng*^*−/−*^, resulted in increased P14 cell proliferation at d8 of acute LCMV infection (Fig. S4 D). Concurrent with this, IFNAR deficiency decreased T_EFF_ cells and increased T_SCM_ cell formation, similar to that observed with d0–1 IFNAR blocking (Fig. 8 D and Fig. S4 E). Unlike *Ifnar*^*−/−*^ mice, T_SCM_ cell formation was promoted in *Ifng*^*−/−*^ hosts in the absence of ongoing viral load, suggesting that the inflammatory regulation of cell position plays a role in addition to increased antigen burden (Fig. 8 E). Combined, these data suggest that for each IFN-deficient model, cell retention in the paracortex and increased T_SCM_ cell formation occurred by two distinct mechanisms. Firstly, the IFNγ-dependent increase of CXCR3 chemokines mediates gradient destruction and CXCR3 receptor internalization in *Ifnar*^*−/−*^ (or d0–1 IFNAR-blocked) lymph nodes. Secondly, the reduction or total ablation of chemokine expression in *Ifng*^*−/−*^ and 2xIFN^−/−^ mice limits T cell migration to the lymph node periphery (Fig. S4 F). In these settings, retention in the paracortex is likely mediated by the TCF-1–regulated expression of CCR7 (Duckworth et al., 2021). Together, these data underscore the importance of chemokine regulation and spatial location for the differentiation of T_SCM_ cells in vivo.

### Type I IFN inhibition promotes almost exclusive differentiation of T_SCM_ cells following mRNA-LNP vaccination

**Figure S5.**
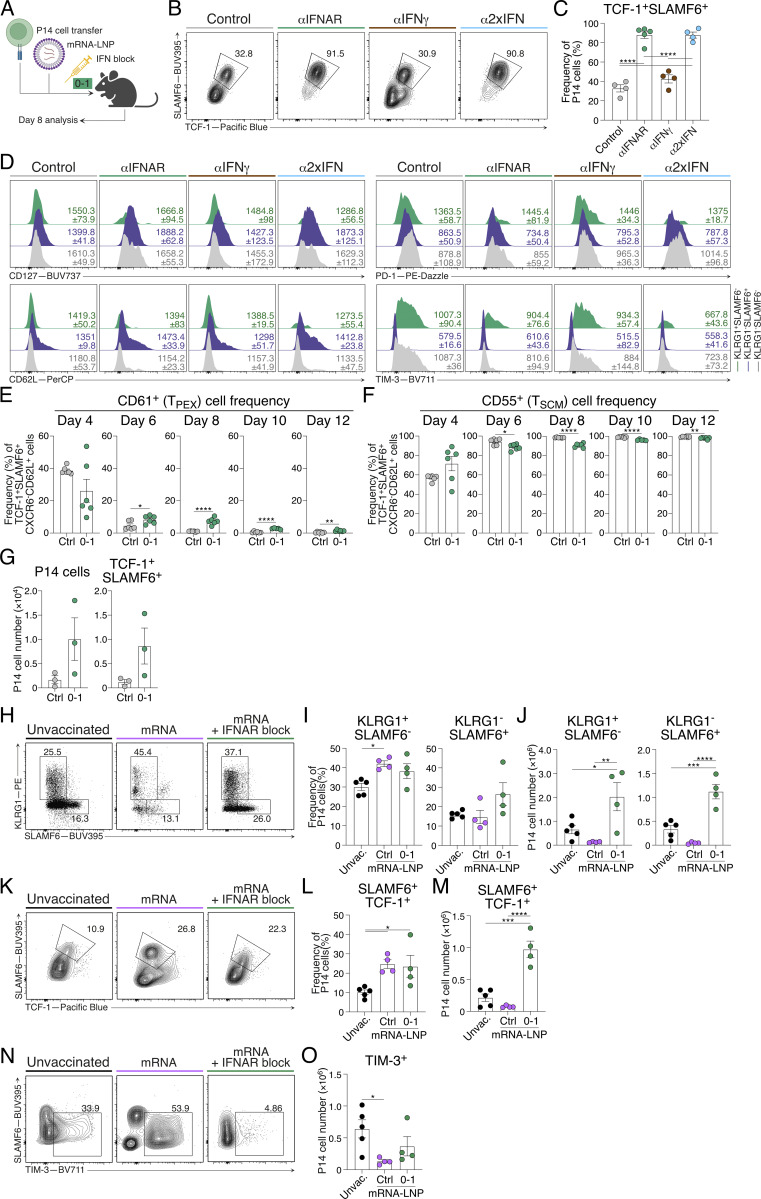
**IFNAR inhibition alongside mRNA-LNP vaccination promotes exclusive generation of T**_**SCM**_ **cells, driving increased proliferation of specific T cells upon viral rechallenge.** Related to Fig. 9. **(A–D)** Draining lymph node P14 cells from mice d8 following GP33-encoding mRNA-LNP vaccination in combination with IFNAR and/or IFNγ blockade at d0–1. Data are representative of two independent experiments with four to five mice per group in each experiment. Each dot in C represents a single mouse. Data are the mean ± SEM. Statistical differences were analyzed using one-way ANOVA tests. **(A)** Experimental scheme. Naïve P14 cells were adoptively transferred into wild-type host mice 24 h prior to mRNA-LNP vaccines, which encode the GP33 epitope. Cohorts did not receive further treatment or received treatments of either IFNAR blocking, IFNγ blocking, or a combination at d0–1 following vaccination. **(B)** Representative flow cytometry plots showing T_SCM_ (TCF-1^+^SLAMF6^+^) cell populations of P14 cells within each condition. **(C)** Graph summarizing frequencies in B. Each dot represents a single mouse sample. **(D)** Representative histograms of T_EFF_ (KLRG1^+^SLAMF6^−^), T_SCM_ (KLRG1^−^SLAMF6^+^), and T_EX_ (KLRG1^−^SLAMF6^−^) P14 cell populations from control, IFNAR-blocked, IFNγ-blocked, or combined IFNAR- and IFNγ-blocked mice for expression of CD127, CD62L, PD-1, and TIM-3. Average gMFI ± SEM for each graph are indicated. **(E and F)** Graphs summarizing (E) CD61^+^CD55^−^ and (F) CD61^−^CD55^+^ P14 cell frequencies. Analysis of P14 cells from days 4 to 12 following mRNA-LNP vaccination. Data are the mean ± SEM. Each dot represents a single mouse. Data are representative of two independent experiments with five to six mice per group. Statistical differences were analyzed using unpaired *t* tests at each time point after vaccination. **(G)** Graphs summarizing the total P14 cell and T_SCM_ (TCF-1^+^SLAMF6^+^) cell numbers within control and d0–1 IFNAR-blocked mice 28 days following mRNA-LNP vaccination. **(H–O)** Comparison of P14 cell response to chronic LCMV infection rechallenge in mice that received d0–1 IFNAR blocking following mRNA-LNP vaccination with unvaccinated mice and mice that received only vaccination as described in Fig. 9 I. **(H)** Representative plots of P14 cells showing T_EFF_ (KLRG1^+^SLAMF6^−^) and T_SCM_ (KLRG1^−^SLAMF6^+^) cell populations. **(I)** Graphs summarizing population frequencies in H. **(J)** Cell counts of T_EFF_ (KLRG1^+^SLAMF6^−^) and T_SCM_ (KLRG1^−^SLAMF6^+^) cell populations in each experimental condition. **(K)** Representative flow cytometry plots of T_SCM_ (TCF-1^+^SLAMF6^+^) P14 cell populations. **(L)** Graph summarizing frequencies in K. **(M)** T_SCM_ (TCF-1^+^SLAMF6^+^) cell counts. **(N)** Representative flow cytometry plots of TIM-3 expression on P14 cells within each condition. **(O)** TIM-3^+^ P14 cell counts. *P < 0.05, **P < 0.01, ***P < 0.001, ****P < 0.0001.

**Figure 9.**
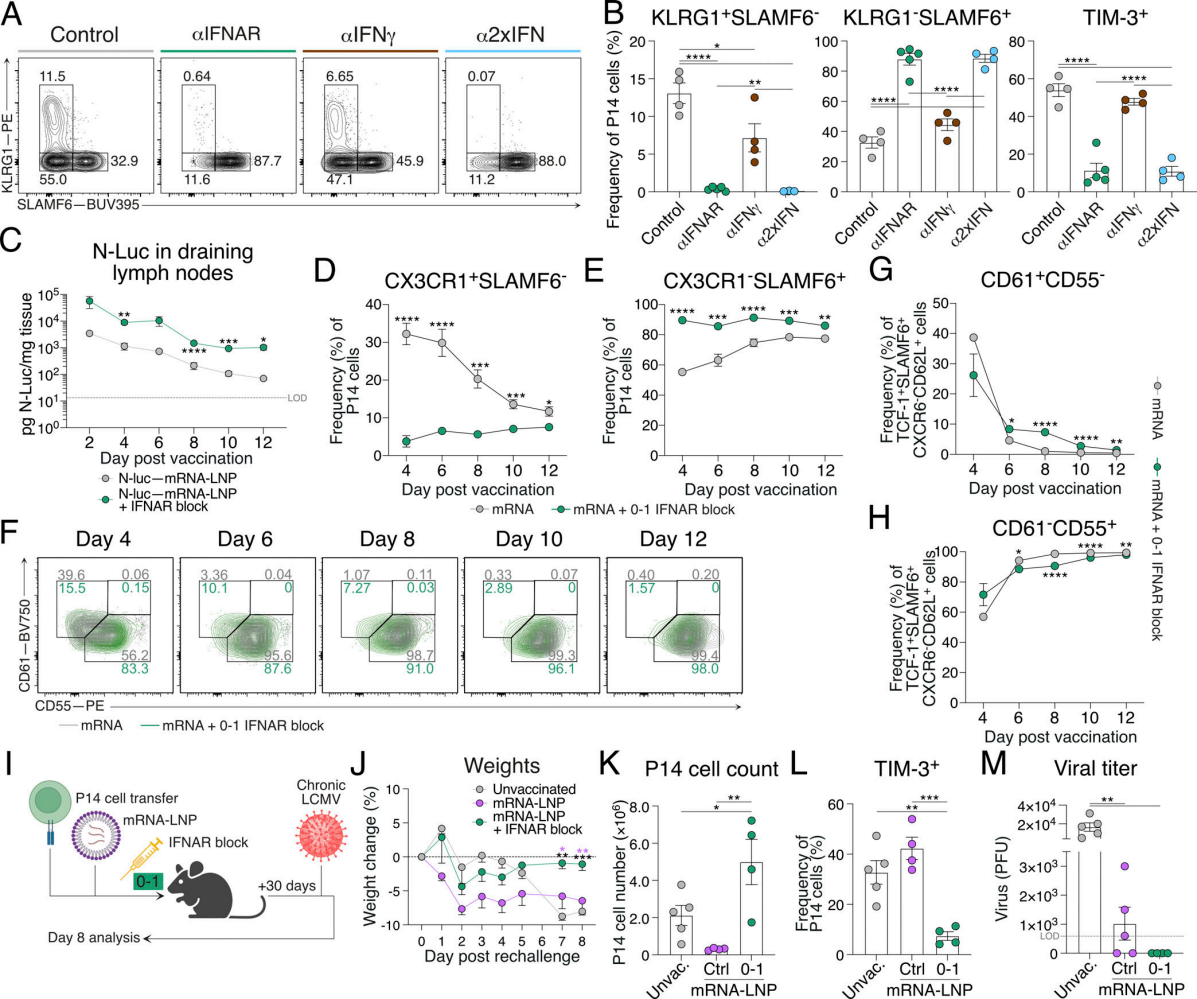
**IFNAR blocking in combination with mRNA-LNP vaccination promotes T**_**SCM**_ **differentiation conferring enhanced protective capacity. (A and B)** P14 cells from mice that received intramuscular vaccinations of GP33-encoding mRNA-LNP and d0–1 treatment of IFNAR and/or IFNγ blocking. Draining lymph node P14 cells were analyzed at d8 following vaccination. Data are representative of two independent experiments with four to five mice per group in each experiment. Each dot in B represents a single mouse. Data are the mean ± SEM. Statistical differences were analyzed using one-way ANOVA tests. **(A)** Representative plots of P14 cells showing T_EFF_ (KLRG1^+^SLAMF6^−^), T_SCM_ (KLRG1^−^SLAMF6^+^), and T_EX_ (KLRG1^−^SLAMF6^−^TIM-3^+^) populations. **(B)** Graphs summarizing frequencies in A. **(C)** Graph summarizing the persistence of antigen (NLuc) within draining lymph nodes from NLuc-encoding mRNA-LNP–vaccinated mice with or without d0–1 IFNAR blocking treatments over time. Data are the mean ± SEM. The dashed line indicates NLuc LOD from blank tissue. **(D–H)** Analysis of antigen-specific P14 cells from days 4 to 12 following mRNA-LNP vaccination. Data are the mean ± SEM. Data are representative of two independent experiments with five to six mice per group. Statistical differences were analyzed using unpaired *t* tests at each time point after vaccination. **(D and E)** Graphs summarizing (D) CX3CR1^+^SLAMF6^−^ (T_EFF_) cells and (E) CX3CR1^−^SLAMF6^+^ (T_SCM_) cells. **(F)** Representative flow cytometry plots of gating CD61^+^ T_PEX_ cells and CD55^+^ T_SCM_ cells. Cells are pre-gated on TCF-1^+^SLAMF6^+^CXCR6^−^CD62L^+^ cells as per Fig. S2 F. **(G and H)** Graphs summarizing (G) CD61^+^CD55^−^ and (H) CD61^−^CD55^+^ cell frequencies. **(I–M)** Comparison of response to chronic LCMV infection rechallenge in mice that received d0–1 IFNAR blocking following mRNA-LNP vaccination with unvaccinated mice and mice that received only vaccination. Data are the mean ± SEM. Each dot in K–M represents a single mouse. Data are representative of three independent experiments with four to five mice per group. Statistical differences were analyzed using one-way ANOVA. **(I)** Schematic illustration of the experiment timeline. P14 cells were transferred into wild-type hosts prior to intramuscular vaccination of GP33-encoding mRNA-LNP. Half of the vaccinated mice immediately received IFNAR blocking followed by secondary treatment a day later. Mice were left for 30 days to establish memory. Another cohort of naïve mice received adoptive cell transfer of naïve P14 cells a day prior to all mice being infected with chronic LCMV. Mice were weighed daily following rechallenge, and lymph nodes were collected at d8. **(J)** Graph summarizing average proportion of weight change over the course of chronic LCMV infection within each group. **(K)** Graph of total P14 cell count in each indicated experimental group. **(L)** Expression of exhaustion marker TIM-3 on the surface of P14 cells. **(M)** Graph summarizing the PFU from viable virus in spleens of mice in each experimental group. The dashed line indicates viral plaque LOD. *P < 0.05, **P < 0.01, ***P < 0.001, ****P < 0.0001. Fig. S5 show additional supporting data. LOD, limit of detection.

As noted, IFNAR blockade alters chemokine expression and promotes T_SCM_ cell differentiation independently of viral burden (Fig. S3 F). In view of this, we reasoned that short-term IFN blockade could be leveraged in a vaccine setting for the promotion of T_SCM_ cell differentiation and enhanced immune memory. While the level of IFN-Is and IFN-II induced between viral and mRNA-LNP vaccine settings is distinct, LNPs have their own adjuvant effect and vaccine-encoded mRNA may still induce IFN-I despite incorporating modified nucleosides into the antigen-encoding mRNA (Alameh et al., 2021; Li et al., 2022). Indeed, in addition to IL-6 production, CXCL10 is also highly upregulated in lymph nodes 4 h following LNP administration, suggesting IFNs may play a role in modulating mRNA-LNP CD8^+^ T cell responses induced by nucleoside-modified mRNA-LNP vaccines (Alameh et al., 2021; Li et al., 2022). To test this, we generated mRNA-LNP vaccines encoding a GP33 minigene recognized by P14 cells. P14 cells were adoptively transferred into mice a day before flank thigh intramuscular vaccinations. Following vaccination, mice received either or combined d0–1 IFNAR and IFNγ blocking antibodies (Fig. S5 A). On d8 following vaccination, mRNA-LNP–induced P14 cell differentiation was dominated by both TCF-1^+^SLAMF6^+^ T_SCM_ cells and TIM-3^+^(KLRG1^−^SLAMF6^−^) T_EX_ cells (Fig. 9, A and B; and Fig. S5, B–D). Distinct from our observations during acute LCMV infection, IFNγ blockade in combination with mRNA-LNP vaccination failed to alter T_SCM_ and T_EX_ differentiation, suggesting a negligible role of IFNγ in T_SCM_ cell formation following vaccination (Fig. 9, A and B; and Fig. S5, B and C). In contrast, d0–1 IFNAR blocking alone (αIFNAR) or in combination with IFNγ blocking (α2xIFN) ablated both T_EFF_ and T_EX_ cell formation, leading to the specific promotion of T_SCM_ cells (Fig. 9, A and B; and Fig. S5, B–D). T_SCM_ cells formed ∼90% of all antigen-specific P14 cells, compared with ∼40% in control-treated and single IFNγ–blocked (αIFNγ) groups. The almost exclusive formation of T_SCM_ cells in response to d0–1 IFNAR inhibition in combination with mRNA-LNP vaccination demonstrates a novel approach to selectively promote vaccine-induced T_SCM_ cell formation.

### Stem-like CD8^+^ T cell conversion occurs in the context of vaccination

To determine whether enhanced vaccine-induced T_SCM_ cell differentiation was associated with increased antigen expression, we analyzed the expression of mRNA-encoded nanoluciferase (NLuc) reporter expression with or without IFNAR blockade. Similar to that seen in viral infection (Figs. 2 A and 4 F), d0–1 IFNAR blockade increased and prolonged antigen expression following mRNA-LNP vaccination (Fig. 9 C). We next investigated whether the transition between T_PEX_ and T_SCM_ cellular states was a natural developmental pathway for memory formation in response to vaccination, as we observed after viral infection (Fig. 4, C–E). For this, we tracked the transition of stem-like CD8^+^ T cell states following GP33 mRNA-LNP vaccination. In this setting, IFNAR blockade resulted in an increased frequency of TCF-1^+^SLAMF6^+^ stem-like CD8^+^ T cells at the cost of CX3CR1^+^ effector cell differentiation after vaccination (Fig. 9, D and E). Within the TCF1^+^SLAMF6^+^ stem-like CD8^+^ T cell population, CD61^+^ T_PEX_ cells were evident at d4 following vaccination and decreased over time, resulting in the complete conversion to CD55^+^ T_SCM_ cells at d8 (Fig. 9, F–H; and Fig. S5, E and F). Following d0–1 IFNAR blockade, this conversion from CD61^+^ T_PEX_ cells was delayed, similar to that observed in acute LCMV infection (Fig. 4), with the complete conversion to CD55^+^ T_SCM_ occurring at day 10 (Fig. 9, F–H; and Fig. S5, E and F). Combined, this suggests that the T_PEX_ cell phenotype is an intermediate cellular state during the generation of T_SCM_ cell memory, broadening the importance of tracking stem-like transition for the establishment of vaccine-induced immune protection.

### IFNAR inhibition with mRNA-LNP vaccination confers superior immune protection

The early generation of T_SCM_ cells with mRNA-LNP vaccination correlates with durability of CD8^+^ T cell responses (Akondy et al., 2017; Baharom et al., 2021; Jung et al., 2022). Indeed, 28 days following a single mRNA-LNP vaccination combined with d0–1 IFNAR resulted in an increased frequency of T_SCM_ cells in the draining lymph nodes compared with control vaccinated mice (Fig. S5 G). We next sought to determine whether our d0–1 IFNAR-blocked mRNA-LNP vaccination protocol would provide improved immune recall. For this, GP33-encoding mRNA-LNP–vaccinated mice were rested for 30 days to establish memory. An additional cohort of unvaccinated mice received cell transfers of naïve P14 cells 1 day prior to all groups being challenged with chronic LCMV (Fig. 9 I). Consistent with chronic infection, all groups exhibited weight loss by d4–5 (Fig. 9 J). While the weights of unvaccinated and control vaccinated mice continued to decrease, mice that received d0–1 IFNAR blockade at the time of mRNA-LNP vaccination rapidly returned to their pre-challenge weight (Fig. 9 J). Despite the frequencies of T_EFF_ and T_SCM_ cell populations being similar between groups, improved recovery in d0–1 IFNAR-blocked mice was associated with an increased lymph node P14 cell number, suggesting increased proliferative burst or increased cell survival (Fig. 9 K and Fig. S5, H–M). Consistent with increased protection, P14 cells within the d0–1 IFNAR-blocked group had reduced TIM-3 expression (Fig. 9 L; and Fig. S5, N and O) and more rapid viral clearance (Fig. 9 M). Collectively, these data demonstrate the application of early IFNAR inhibition in a vaccine setting to drive the selective generation of T_SCM_ cells for enhanced immune memory.

## Discussion

The promotion of TCF-1^+^ T_SCM_ cells is a major goal of prophylactic vaccines that elicit T cell memory and cancer therapeutic vaccines that promote tumor clearance (Held et al., 2019; Lugli et al., 2020; Zhao et al., 2022). Here, we propose that early IFN-I blockade at the time of T cell priming optimizes the formation of TCF-1^+^ T_SCM_ cells in vivo. Given the essential role IFN-I plays during antiviral responses, it may seem counterintuitive to block this pathway to enhance immune protection (Crouse et al., 2015; Lazear et al., 2019). Indeed, previous work has demonstrated that IFNAR deficiency or extended blocking of IFNAR establishes persistent viral load and chronic-like infection (Teijaro et al., 2013; Wilson et al., 2013). Our study differs from this previous work by limiting the IFNAR blockade to the initial days of infection. We show that blocking IFNAR during this window extends the time of viral persistence; however, viral load is cleared within 14 days. Further, we show that the requirement for IFN-I to drive T_EFF_ cell differentiation is not relevant in vaccine settings, where there is no pathogen to overcome.

Our study clarifies the distinction between T_SCM_ and T_PEX_ stem-like cellular states, inferring that the presence of antigen load discriminates these two states and that once cleared, T_PEX_ cells transition into T_SCM_ cells to maintain the memory pool. We established and validated a method to monitor the transition between transcriptionally distinct T_PEX_ and T_SCM_ cell states to enable the tracking of these individual cellular states during vaccination, infection, and immunotherapy. Of note, CD55 marks self-renewing cells in other systems and both CD55 and CD61 mediate cell adhesion, suggesting transition between these cellular states may alter cellular interactions (Cimmino et al., 2016; Saygin et al., 2017; Zhang et al., 2022). These findings emphasize the duality of T_PEX_ cell potential to not only generate effector and terminally exhausted cells in chronic settings (Beltra et al., 2020; Chen et al., 2019; Daniel et al., 2022; Hudson et al., 2019; Utzschneider et al., 2016), but also transition into a potent memory population when antigen is cleared. Thus, the T_PEX_ cellular state appears to reflect a natural precursor of T_SCM_ cells and this transition is interrupted in the presence of chronic viral load (Chu et al., 2025; McManus et al., 2025; Utzschneider et al., 2020). Further, when reexposed to antigen, T_SCM_ cells can revert back to T_PEX_ cells, which may endower the superior proliferative and protective potential of this memory population (Akondy et al., 2017; Fuertes Marraco et al., 2015; Gattinoni et al., 2011; Jeannet et al., 2010; Pais Ferreira et al., 2020; Zhou et al., 2010). Together, this establishes a fluid developmental relationship between stem-like cellular states that is dependent on the antigenic environment and suggests that monitoring stem-like cell conversion could act as a biomarker for disease progression and regression during chronic infection and for the establishment of memory following vaccination and immunotherapy.

Our results reveal an underappreciated interplay between IFN-I inhibition and increased IFN-II production that was identified through investigation of chemokine biology. During viral infection and autoimmune disease, IFN-I– and IFN-II–induced signatures overlap and are thus difficult to dissect (Lazear et al., 2019; Rusinova et al., 2013; Walker et al., 2021). We reveal that IFN-I inhibition leads to an unexpected increase in IFN signature chemokines. Here, we have focused on the expression of CXCL9 and CXCL10, as C57BL/6 mice lack a functional CXCL11 protein, and when CXCL11 is present, there is no overt impact on CD8^+^ T cell differentiation in LCMV infection (Dalit et al., 2022). Increased *Cxcl9* and *Cxcl10* are in line with observations during infection and vaccination where IFN-I signaling is suppressed and chemokine transcription remained elevated (Blanco-Melo et al., 2020; Borriello et al., 2022; Hoagland et al., 2021). The increased chemokine expression was due to IFNγ, as double deficient (2xIFN^−/−^) mice failed to induce *Cxcl9* and *Cxcl10*. The increase in IFNγ is likely due to an increase in the number of IFNγ^+^ NK cells, which may act to generate a feed-forward inflammatory loop to increase *Cxcl9* expression (Groom and Luster, 2011b). These results propose a re-evaluation of the influence of IFNγ in studies investigating roles of IFN-I, via IFNAR blocking or deficiency, in vaccine and viral responses and have implications for individuals with inborn errors in IFN-I signaling or neutralizing IFN-I autoantibodies (Bastard et al., 2020; Casanova and Abel, 2022; Sposito et al., 2021; Zhang et al., 2020).

We showed that the promotion of T_SCM_ cells by IFNAR inhibition was indirect to intrinsic CD8^+^ T cell IFNAR signaling. This is in contrast to previous studies that explored the role of IFNAR directly in CD8^+^ T cells for survival and effector formation (Crouse et al., 2015; Lazear et al., 2019). The promotion of T_SCM_ cells was associated with increased antigen load and was observed for both viral infection and mRNA-LNP vaccination. This is consistent with the notion that increased vaccine reservoirs enhance immune protection (Lee et al., 2022). An important aspect of our study was to investigate the lymph node microenvironment and mechanism by which chemokine gradients direct T cell positioning. We demonstrate T_SCM_ cell differentiation is promoted by both low expression and overexpression of CXCR3 chemokines. In each of these conditions, chemokine gradients were disrupted and lead to an increase of CD8^+^ T cells in the lymph node paracortex. To our knowledge, this is the first in vivo demonstration where inflammation leads to the abundance of chemokine, which disrupts chemokine gradient formation and restricts cell migration. Our observations are consistent with the analysis of in vitro migration assays where desensitization of receptors limits migration potential (Lammermann and Kastenmuller, 2019). This phenomenon of in vivo chemokine abundance regulating cell position may be relevant to other settings including lymphocyte congestion that is observed in the deficiency of atypical chemokine receptors or overexpression of chemokines within the tumor microenvironment (Lee et al., 2011; Tan et al., 2018). Although it is not fully resolved why paracortex positioning benefits stem-like CD8^+^ T cell differentiation, we propose that chemokine desensitization alters the dynamics of T cell priming within the T cell paracortex where CXCL9-expressing cDC1 cells reside (Broomfield and Groom, 2024; Bullock et al., 2024; Duckworth and Groom, 2021; Duckworth et al., 2022; Palacio et al., 2020). Supporting this, previous work has shown that targeting antigen toward cDC1s promotes the differentiation toward a memory CD8^+^ T cell phenotype and that cDC1s form a protective niche for stem-like memory CD8^+^ T cells (Broomfield and Groom, 2024; Caminschi et al., 2008; Cheang et al., 2024; Dahling et al., 2022).

Our results have significant implications for vaccination and adjuvant approaches that induce IFN-I and IFN-II, suggesting that in order to promote CD8^+^ T cell memory formation, this induction should be limited (Alameh et al., 2021; Arunachalam et al., 2021; Borriello et al., 2022; Li et al., 2022; Quinn et al., 2015). We note that early IFN-I blockade decouples T_SCM_ differentiation and cell expansion, similar to other studies where T_SCM_ cells are promoted (Gautam et al., 2019). This is a particularly beneficial outcome in the context of vaccination to further increase the pool of memory T cells. Early IFNAR inhibition in combination with mRNA-LNP vaccination promotes T_SCM_ cell formation and provides a protective benefit against chronic LCMV infection, compared with mRNA-LNP vaccination alone. This resembles the delayed IFN-I response observed following the YF-17D yellow fever vaccine, which induces a robust and stable T_SCM_ cell population and decades-long protection (Ahmed and Akondy, 2011; Akondy et al., 2017; Hagan et al., 2022; Pais Ferreira et al., 2020). Supporting this notion, vaccine responses in individuals with inborn errors in IFN-I signaling or neutralizing IFN-I autoantibodies demonstrate this is unlikely to come at a cost to humoral immunity (Sokal et al., 2023). The ability to pharmacologically induce TCF-1^+^ T_SCM_ cells suggests that it is feasible to design vaccines to establish long-term prophylactic protection and for personalized cancer vaccines with the potential to overcome insensitivity to PD-1 blockade (D'Alise et al., 2022; Held et al., 2019; Lugli et al., 2020; Rojas et al., 2023; Siddiqui et al., 2019; Zhao et al., 2022). Combined, this study contributes to the basic understanding of the processes leading to T cell memory and applies this knowledge to reveal a promising approach to increase immune protection following vaccination.

## Materials and methods

### Mice

Mice were bred and maintained on a C57BL/6 background under specific pathogen–free conditions. REX3-transgenic (Groom et al., 2012), *Ifnar*^−/−^ (Hwang et al., 1995), *Ifng*^−/−^ (Dalton et al., 1993), *Cxcl9*^−/−^ (Park et al., 2002), *Cxcl10*^−/−^ (Dufour et al., 2002), P14-transgenic (Pircher et al., 1989), and Ly5.1 (Shen et al., 1985) mice have been previously described. REX3 mice were bred with *Ifnar*^−/−^ and/or *Ifng*^−/−^ to generate REX3 *Ifnar*^−/−^, REX3 *Ifng*^−/−^, and REX3 *Ifnar*^−/−^*Ifng*^−/−^ mice. Mice with GFP under the chicken β-actin promoter (Schaefer et al., 2001) were bred with P14 and Ly5.1 mice. All experiments were conducted in compliance with the Walter and Eliza Hall Institute (WEHI) Animal Ethics Committee and performed on mice of 6–10 wk of age of mixed sex.

### Adoptive cell transfer, viral infection, and IFN inhibition

Naïve P14 cells were isolated using Naïve CD8a^+^ T-cell Isolation Kit (Miltenyi Biotec), and 2 × 10^4^ cells were transferred into host mice via intravenous lateral tail injection 1 day prior to viral infection. Mice were inoculated with 3 × 10^4^ or 2 × 10^6^ plaque-forming units (PFU) of acute LCMV Armstrong or chronic LCMV Docile, respectively, via intravenous lateral tail injection. Unless indicated otherwise, mice received two (d0–1) intraperitoneal injections of either or combined 200 μg anti-IFNAR monoclonal antibodies (clone MAR1-5; Leinco Technologies, and gift from Paul J. Herzog, Hudson Institute of Medical Research, Clayton, Australia) and 200 μg anti-IFNγ monoclonal antibodies (WEHI Antibody Facility).

### Preparation of samples for flow cytometry

Blood samples were twice treated with red cell lysis buffer (WEHI Media) for 3 min at room temperature to remove red blood cells. Single-cell suspensions were stained for surface antigen expression using indicated antibodies for 20 min at 4°C, followed by viability dye staining for 10 min at 4°C. Transcription factor and chemokine stainings were performed using Foxp3 Transcription Factor Staining Kit (Thermo Fisher Scientific). FlowSOM analysis defined cell clusters based on differential staining of CXCR3, CD127, CD62L, PD-1, SLAMF6, CXCR6, TIM-3, CXCR5, CD101, KLRG1, CX3CR1, SCA-1, and CD44. All flow cytometry analysis was performed on BD FACSymphony A3 Cell Analyzer (BD Biosciences) and Cytek Aurora (Cytek Biosciences), and data were analyzed using FlowJo v10 (FlowJo LLC) and the FlowSOM plugin.

### Viral titer of tissues

Spleen tissues were harvested and homogenized using Qiagen TissueLyser for 7 min at 30 Hz. LCMV viral titers were determined using a focus-forming assay, as previously outlined (Battegay et al., 1991).

### Preparation of samples for cell sorting and scRNAseq

Lymph nodes were harvested and processed to form a single-cell suspension. Samples were stained for surface antigen expression using indicated antibodies and TotalSeq hashtags (BioLegend) for 30 min at 4°C, followed by viability dye staining for 10 min at 4°C. Sample suspensions were sorted using BD FACSAria Fusion Flow Cytometer (BD Biosciences) to isolate antigen-specific P14 CD8^+^ T cells.

### Data processing and demultiplexing of scRNAseq data

For data processing, reads from each capture were processed using 10X Genomics Cell Ranger software (v7.0.0). Firstly, “cellranger mkfastq” and bcl2fastq (v2.19.1) were used to convert and demultiplex Illumina sequencer’s BCL files into FASTQ files for each of the gene expression (GEX), antibody-derived tag (ADT), and hashtag oligo (HTO) libraries. Secondly, “cellranger multi” was used with default settings to generate count matrices. The GEX data were mapped and quantified against the 10X Genomics pre-built mm10 (vM23/Ensembl 98; GENCODE) reference groups and transcriptome (2020-A [July 7, 2020] version), and the feature barcoding (ADT and HTO) data were quantified against a “feature.csv” file containing the barcode sequences provided by BioLegend. Finally, the DropletUtils R/Bioconductor package (v1.18.1) was then used to load the Cell Ranger output files into R (v4.2.1) and to identify non-empty droplets using the “emptyDrops” method with default settings (Lun et al., 2019). For demultiplexing, the “demuxmix” method from the demuxmix (v1.0.0) R/Bioconductor package was applied to the HTO data, with the “naive” model and default parameters, to multiplex non-empty droplets to their sample of origin. This was performed separately for each capture. A droplet was assigned to a sample if the best demuxmix assignment matched the corresponding HTO combination of a sample or was otherwise assigned as a “multiplet,” “negative,” or “uncertain” sample. All scripts used are available from https://github.com/WEHISCORE/G000304_Broomfield.

### Analysis of scRNAseq and cellular indexing of transcriptomes and epitopes by sequencing (CITEseq) data

#### Quality control (QC) and clustering

QC on the demultiplexed scRNAseq data was conducted using the scater R package (McCarthy et al., 2017) to remove low-quality cells and cells with high mitochondrial genes (≥10%), leaving 17,629 cells for analysis. The data were then normalized using SCTransform in the Seurat R package (Hao et al., 2021) and clustered using the default Louvain method at resolution 0.8, resulting in 15 clusters. Curation based on proportion distribution by conditions led to the merging of two clusters, resulting in 14 clusters for downstream analysis. Module scores of each cluster were calculated using the “AddModuleScore” function in Seurat based on the anchor genes selected from previous studies for T_SCM_, T_PEX_, and T_EX_ cells (Fig. S1 A) (Gattinoni et al., 2011; Fuertes Marraco et al., 2015; Im et al., 2016; Utzschneider et al., 2016; Akondy et al., 2017; Sade-Feldman et al., 2018; Siddiqui et al., 2019; Beltra et al., 2020; Andreatta et al., 2021; Baharom et al., 2021; Connolly et al., 2021).

#### Differential expression (DE) analysis

DE was conducted using a pseudobulk approach based on “cluster” and “samples,” and after filtering out samples with <10 cells and applying gene-level QC using edgeR::filterByExpr, leaving in 131 pseudosamples and 9,438 genes for analysis. DE analyses were conducted using a *voom-limma-duplicatecorrelation with sample weights* pipeline via the *edgeR::voomLmFit* function to fit a linear model with “condition_cluster” (condition being infectious setting) as the covariate, and to estimate the consensus correlation across mice and account for mouse variation as a random effect (Robinson et al., 2010; Ritchie et al., 2015). DE was conducted for the following comparisons based on four clusters identified from the cluster analysis: (1) d8 acute LCMV C8 versus all other clusters, (2) d8 chronic LCMV C2 versus all other clusters, (3) d8 IFNAR-blocked acute LCMV C2 versus all other clusters, (4) d14 IFNAR-blocked acute LCMV C0 versus all other clusters, and (5) clusters (d8 chronic LCMV C2 + d8 IFNAR-blocked acute LCMV C2) versus clusters (d14 IFNAR-blocked acute LCMV C0 + d8 acute LCMV C8). An empirical Bayes moderated t-statistic was generated with multiple testing adjustment carried out using the Benjamini–Hochberg procedure to identify statistically significant genes (adjusted P < 0.05). The enrichment of marker gene sets from published datasets (Akondy et al., 2017; Baharom et al., 2021; Beltra et al., 2020; Chen et al., 2019; Gattinoni et al., 2011; Ivanova et al., 2002; Luckey et al., 2006; Sade-Feldman et al., 2018; Siddiqui et al., 2019; Utzschneider et al., 2016) in the DE results was calculated using the *fgsea* package (Korotkevich et al., 2021, *Preprint*) and visualized as NES dot plots.

#### Data processing and visualization of CITEseq analysis

CITEseq data were extracted and normalized using the centered log-ratio transformation method across cells from the *Seurat::NormalizeData* function. The log count expression for selected markers was visualized by overlaying onto the scRNAseq UMAPs of CD8^+^ T cells (Stuart et al., 2019).

### DC isolation from lymph nodes

Lymph nodes were mechanically disrupted with tweezers and incubated in RPMI with 0.8 mg/ml Dispase II (Roche), 0.2 mg/ml collagenase P (Roche), and 0.1 mg/ml DNase I (Sigma-Aldrich) for 20 min at 37°C. The supernatant was collected, and remaining tissue pieces were further incubated twice more in fresh digestion medium for 10 min at 37°C each.

### Whole-tissue immunofluorescence staining, clearing, and LSFM

Lymph nodes were harvested and fixed in 4% paraformaldehyde (Sigma-Aldrich) for 12–24 h at 4°C, followed by incubation in blocking buffer containing 1% bovine serum albumin (Sigma-Aldrich), 1% normal rat serum (Jackson ImmunoResearch), and 0.3% Triton X-100 (Sigma-Aldrich) in PBS for 24 h at 4°C. Whole tissues were stained with indicated antibodies in blocking buffer for 3 days at 4°C. Tissues were washed by immersion in PBS containing 0.5% 1-thioglycerol (Sigma-Aldrich) and 0.2% Triton X-100 (Sigma-Aldrich) for 24 h at room temperature. Lymph nodes were immersed in Ce3D clearing medium containing 1.455 g/ml Histodenz (Sigma-Aldrich), 40% N-methylacetamide (Sigma-Aldrich), 0.5% 1-thioglycerol, and 0.1% Triton X-100 in PBS for 24 h at room temperature on a shaking incubator (Li et al., 2017). The clearing medium was replaced with fresh medium, and tissues were incubated for a further 2–3 days to clear the lymph nodes to a refractive index of 1.49–1.5. The cleared lymph nodes were embedded in 2% low-melting agarose (Sigma-Aldrich) containing 1:10,000 Fluoresbrite YG microspheres 1 µm (Polysciences) in 2.15-mm-diameter glass capillaries (Zeiss). Samples were submerged in clearing solution for 24 h before imaging to allow for refractive index matching between the agarose and clearing medium. For LSFM, images were acquired on a Z.1 light-sheet microscope (Zeiss) using a 5× (f/0.16) air objective. LSFM images were processed using ZEN Blue and ZEN Black (Zeiss) and Imaris 9.7.2 (Oxford Instruments). Pseudocolor FIRE intensity LUT scales were set to the brightest pixel intensity across samples and maintained for all comparisons for each reporter protein.

### Quantification of cells in intact lymph nodes

Analyses were performed using the EVF implemented within 3D ImageJ Suite v2.1.0/1.53 (Duckworth et al., 2021; Ollion et al., 2013). First, lymph node images were smoothed using a 3D median filter (with radius rx = 4, ry = 4, and rz = 2). 3D lymph nodes were then segmented using a manually set global thresholding. Images containing cell signals were filtered with a 3D median filter (with radius rx = 2, ry = 2, and rz = 1) followed by a top-hat filtering (with radius rx = 6, ry = 6, and rz = 3) to enhance the signal of spots. The cell signal was manually set using global thresholding. The positioning of cells within the lymph nodes was assessed using an EVF analysis, whereby a 3D distance map was computed inside the lymph node, and then, the distance values were sorted and normalized from 0 to 1. The EVF values were then divided into 100 layers of equal volumes from 0 (near the lymph node periphery) to 1 (lymph node center), and the volume of cells within each layer was computed.

### Migration assay

Axillary, brachial, and inguinal lymph nodes were harvested and mashed into single-cell suspension. Then, 4 × 10^6^ total cells were added to 96-well transwells (Corning Costar) in RPMI with 0.5% FCS and migrated toward 1–10,000 ng/ml CXCL10 chemokine (PeproTech) for 1.5 h. Cells in the lower chamber were collected and stained. Migration of P14 cells was detected by timed acquisition on BD FACSymphony A3 Cell Analyzer (BD Biosciences). The migration index was defined as the percentage of migrated cells relative to the input sample.

### IFNγ cytokine bead array on lymph node tissue lysates

Lymph nodes were harvested from d4 infected mice and snap-frozen on dry ice. The tissue was weighed and digested with 10 μl mg^−1^ DISC lysis buffer (20 mM Tris-HCl pH 7.5, 150 mM NaCl, 2 mM EDTA, 1% Triton X-100, 10% glycerol, H_2_O, protease inhibitor cocktail tablet [Roche]). The tissue was lysed with TissueLyser (85300; Qiagen), and the supernatant was collected between the pellet and fat layers following a 20,000 *g*, 15 -min spin. The supernatant from the tissue lysate was diluted 1:10 and loaded onto a BD Cytometric Bead Array Mouse IFNg Flex set (BD Biosciences), used as per the manufacturer’s instructions. Briefly, tissue lysates and mouse IFNg standards were added to IFNg cytokine capture beads and PE detection reagent in a 96-well plate for 2 h at room temperature, protected from light. The plate was washed two times in BD CBA wash buffer, resuspended, and acquired on a BD LSRFortessa X-20 cell analyzer (BD Biosciences). Data analysis and standard curve generation were performed with FCAP Array Software version 3.0 (BD Biosciences).

### mRNA-LNP vaccine production and vaccination

#### GP33-encoding mRNA-LNP

mRNA production was performed as described previously (Freyn et al., 2020). Briefly, the sequence of the P14 minigene was codon-optimized, synthesized (GenScript), and cloned into an mRNA production plasmid. The mRNA was produced from the linearized plasmid to contain 101 nucleotide-long poly(A) tail. m1Ψ-5′triphosphate instead of UTP was used to generate modified nucleoside-containing mRNA. Capping of the in vitro–transcribed mRNA was performed co-transcriptionally using the trinucleotide cap1 analog, CleanCap (TriLink). mRNA was purified by cellulose purification, as described previously (Baiersdörfer et al., 2019). The minigene-encoding mRNA was analyzed by agarose gel electrophoresis and was stored frozen at −20°C. The cellulose-purified m1Ψ-containing mRNA was encapsulated in LNP using a self-assembly process as previously described wherein an ethanolic lipid mixture of ionizable cationic lipid, phosphatidylcholine, cholesterol, and polyethylene glycol–lipid was rapidly mixed with an aqueous solution containing mRNA at acidic pH (Maier et al., 2013). The LNP formulation used in this study is proprietary to Acuitas Therapeutics; the proprietary lipid and LNP composition are described in US Patent US10,221,127. The mRNA-loaded particles were characterized and subsequently stored at −80°C at an RNA concentration of 1 μg μl^−1^. The mean hydrodynamic diameter of mRNA-LNP was ∼80 nm with a polydispersity index of 0.02–0.06 and an encapsulation efficiency of ∼95%.

For vaccination, mice received a single intramuscular caudal thigh injection of 10 µg GP33 minigene (MKAVYNFATM)-encoding mRNA-LNP prior to intraperitoneal injections of either or combined 200 µg anti-IFNAR or 200 µg anti-IFNγ (WEHI Antibody Facility) monoclonal antibodies.

#### NLuc-encoding mRNA-LNP

The mRNAs used in this study were produced using HiScribe T7 ARCA mRNA Kit (NEB) with linearized double-stranded DNA, generated by PCR amplification, encoding the mRNA sequence, including the ORF of NLuc. Nucleoside-modified mRNA was synthesized using N^1^-methyl-pseudoUTP instead of UTP, with a 5′-Cap 1 structure (TriLink) and in the presence 0.8 M urea. The length of the mRNA was analyzed using denaturing formaldehyde gel electrophoresis, loading 500–1,000 ng of mRNA, and samples were stored at −80°C until further use. The concentration of mRNA was determined using a NanoDrop spectrophotometer (Thermo Fisher Scientific). A lipid mixture of LNPs was prepared by mixing cationic lipid (DLin-MC3-DMA), cholesterol, helper lipid distearoylphatidylcholine (DSPC), and PEGylated lipid (DMG-PEG 2000) at a molar ratio of 50:10:38.5:1.5 into absolute ethanol. In a separate vial, mRNA was dissolved in 30 mM acetate buffer, pH 4, at a concentration of 180 µg ml^−1^. A nitrogen-to-phosphate ratio of 6 was used to formulate the ionizable cationic lipid and mRNA content of LNPs. The aqueous and lipid phases were mixed using NanoAssemblr Ignite utilizing an NxGen microfluidic cartridge (Precision NanoSystems), at a flow rate ratio of 3:1, aqueous:lipid (vol/vol, respectively), and a total flow rate of 4 ml min^−1^. The resulting aqueous/lipid mixture was collected, then diluted by a factor of 3 in Trizma hydrochloride solution (Tris), 25 mM, pH 7.4, and subsequently purified by dialysis against 500 vol of 25 mM Tris, pH 7.4, using Slide-A-Lyzer Dialysis Cassette G2 (15 ml capacity, 10,000 Da; MWCO) for 16–20 h at room temperature. For storage, LNPs were concentrated into 25 mM Tris/8.8% (wt/vol) sucrose (pH 7.4) to achieve 200 ng µl^−1^. The solution was then filtered through a sterilized syringe filter (Millex Low Protein Binding Durapore, 13 mm, polyvinylidene difluoride (PVDF) membrane, 0.22 µm). Finally, LNP particle size and distribution were characterized using dynamic light scattering measurements using a Zetasizer Nano ZS ZEN3600 particle size analyzer at 35 µg ml^−1^ mRNA.

### NLuc quantification

The tissues were isolated and were stored at −80°C immediately after isolation. First, each tissue sample was weighed and recorded. The isolated tissues were then homogenized using gentleMACS Dissociator and suspended in 1 ml Glo lysis buffer 1×, to prepare the tissue lysates. Nano-Glo Dual-Luciferase Assay (Promega) was used to determine the Luc from tissues. In a black 96-well plate containing 50 µl of homogenate tissue in each well, 50 µl NLuc assay reagent (1 in 50 dilution of Nano-Glo Dual-Luciferase Assay substrate into Nano-Glo Luciferase assay buffer) was added. After 15 min, the relative luminescence unit (RLU) was measured by the Envision plate reader at a wavelength of 460 nm. RLU values were converted and normalized to the amount of the Luc protein.

### Quantification and statistical analysis

Statistical differences between groups in datasets with one categorical variable were evaluated by unpaired *t* tests (two groups) or one-way ANOVA (>2 groups) corrected for multiple comparisons. Statistical differences between groups in datasets with two categorical variables were evaluated by two-way ANOVA corrected for multiple comparisons. Exact P values are given for statistical differences between P < 0.05 and 0.0001. All experimental data are presented as the mean ± standard error of the mean (SEM) with statistical analysis performed using Prism 8 (GraphPad Software).

### Online supplemental material

This article includes five supplemental files that show flow cytometry gating strategies, representative plots, and supplemental experimental figures. Fig. S1, relating to Figs. 1 and 2, shows CD8^+^ T cell profiling analysis. Fig. S2, relating to Figs. 3 and 4, shows scRNAseq and scCITEseq (scCITEseq) analysis and CD8^+^ T cell transfer profiling. Fig. S3, relating to Fig. 5, shows DC chemokine analysis and inactivated virus analysis. Fig. S4, relating to Figs. 7 and 8, shows DC chemokine analysis, CD8^+^ T cell profiling, and a model for chemokine-directed cell location. Fig. S5, relating to Fig. 9, shows CD8^+^ T cell profiling following vaccination. Videos 1 and 2 show representative 3D-rendered LSFM of cleared lymph nodes. Table S1 is provided containing all scRNAseq signatures to identify shared and individual transcripts of T_PEX_ and T_SCM_ cell signatures.

## Supplementary Material

Table S1 shows identification of T_PEX_ and T_SCM_ signatures.

## Data Availability

Data are available in the article itself and its supplementary materials. scRNAseq data and scCITEseq data have been deposited at GEO under accession no. GSE289245 and are publicly available as of the date of publication. All experimental models and reagents will be made available upon installment of a material transfer agreement. Further information and requests for resources and reagents should be directed to and will be fulfilled by the lead contact, J.R. Groom (groom@wehi.edu.au; @groomlab).
